# The Role of the Hypoxia-Related Unfolded Protein Response (UPR) in the Tumor Microenvironment

**DOI:** 10.3390/cancers14194870

**Published:** 2022-10-05

**Authors:** Sylwia Bartoszewska, James F. Collawn, Rafal Bartoszewski

**Affiliations:** 1 Department of Inorganic Chemistry, Medical University of Gdansk, 80-416 Gdansk, Poland; 2 Department of Cell, Developmental and Integrative Biology, University of Alabama at Birmingham, Birmingham, AL 35294, USA; 3 Department of Biophysics, Faculty of Biotechnology, University of Wroclaw, F. Joliot-Curie 14a Street, 50-383 Wroclaw, Poland

**Keywords:** ER-stress, hypoxia-reoxygenation injury, TME, cell fate determination, UPRmt

## Abstract

**Simple Summary:**

The complex signaling networks that different cancers utilize for cell survival remain poorly understood. A major problem is the complexity of the tumor microenvironments (TME). Here, we discuss the role of intermittent hypoxia as one of the inducers of the UPR in the TME and the related implications of it for both cancer progression and therapeutic approaches.

**Abstract:**

Despite our understanding of the unfolded protein response (UPR) pathways, the crosstalk between the UPR and the complex signaling networks that different cancers utilize for cell survival remains to be, in most cases, a difficult research barrier. A major problem is the constant variability of different cancer types and the different stages of cancer as well as the complexity of the tumor microenvironments (TME). This complexity often leads to apparently contradictory results. Furthermore, the majority of the studies that have been conducted have utilized two-dimensional in vitro cultures of cancer cells that were exposed to continuous hypoxia, and this approach may not mimic the dynamic and cyclic conditions that are found in solid tumors. Here, we discuss the role of intermittent hypoxia, one of inducers of the UPR in the cellular component of TME, and the way in which intermittent hypoxia induces high levels of reactive oxygen species, the activation of the UPR, and the way in which cancer cells modulate the UPR to aid in their survival. Although the past decade has resulted in defining the complex, novel non-coding RNA-based regulatory networks that modulate the means by which hypoxia influences the UPR, we are now just to beginning to understand some of the connections between hypoxia, the UPR, and the TME.

## 1. Introduction

The tumor microenvironment (TME) is a dynamic network that is created by blood vessels, lymphatic vessels, fibroblasts, immune cells as well as components such as the extracellular matrix (ECM) [1,2] that establishes a “friendly ecosystem” for cancer cells. Since tumors and the selective conditions that are present in the TME influence each other to either promote or to repress cancer progression, understanding the molecular pathways governing these interactions may contribute to the development of novel therapies. During rapid tumor progression, cancer cells are often dealing with hypoxic conditions that are caused a limited blood supply [3,4]. Hypoxia induces a cellular adaptive response that elevates the expression of the transcription factors called hypoxia-inducible factors (HIFs) that activate the global gene expression changes in both non-malignant and cancer cells. HIF-1 and HIF-2 promote increases in the lymphangiogenic and angiogenic responses as well as metabolic changes that lead to a shift to glycolysis [5,6,7,8,9,10,11,12,13,14]. While these transcriptional changes enhance the tumor growth and viability, they also offer potential targets for new cancer therapeutic strategies [14,15,16,17,18,19,20,21,22,23,24,25,26,27,28,29].

While most of the studies in this area have focused on the canonical responses to hypoxia, a better understanding is needed for the complex molecular changes that are found in the hypoxic TME. These changes include the deregulation of endoplasmic reticulum (ER) homeostasis, and the subsequent perturbations in protein folding and secretion [6,30,31,32,33,34,35,36]. The potential for erratic protein folding can also lead to another specialized stress response signaling pathway called the unfolded protein response (UPR) [37]. The UPR promotes survival during hypoxia by restoring the endoplasmic and mitochondrial homeostasis [38,39,40], but at times, it can also inhibit the cancer cell’s survival [41,42,43,44,45,46].

The maturation of transmembrane and secretory proteins [47,48,49,50,51,52,53,54,55] that include proangiogenic receptors and ligands as well as ECM remodeling enzymes [56,57] takes place in the ER [58,59,60]. The ER is also responsible for the assembly of MHC complexes, and consequently, the antigen presentation and immune responses [36,61,62]. Changes in the ER that are caused by hypoxia and the UPR are both important modulators of the TME as well as other homeostatic pathways. Therefore, understanding the crosstalk between hypoxia and the UPR remains critical for understanding the distinction between the cell viability and cell death. Interestingly, although each of these stress responses pathways has been extensively studied individually, the consequences of mutual crosstalk between them remain underappreciated and poorly understood [46]. In this review, we summarize and discuss the implications of the hypoxia-related UPR on the TME components and the ways in which they affect cancer progression.

## 2. Hypoxia as an Activator of the UPR in the Tumor Microenvironment

Both hypoxia and the persistent deregulation of ER homeostasis during the activation of the UPR have been reported to be important features of the TME that affect both cancerous as well as non-malignant cells [31,46,59,60,63,64,65,66,67,68,69,70,71,72,73,74]. The constitutive activation of these pathways supports cancer cell survival through proliferation and by altering the innate and adaptive immune cells to promote the tumor’s progression and metastasis. Although the unmet oxygen demand does not dramatically restrict the disulfide bond formation during the protein synthesis, the posttranslational folding of the proteins is oxygen-dependent. Hypoxia limits the activity of the oxygen-dependent ER-localized oxidoreductase (ERO1α), and this leads to the deregulation of the posttranslational protein modifications and thereby, promotes ER stress [75,76]. Furthermore, exposure to hypoxia often results in the alternative splicing of several common proteins that can lead to the activation of UPR signaling [77]. Notably, the lipid desaturation processes that are necessary for maintaining ER membrane homeostasis are oxygen-dependent as well [78].

The cellular oxygen levels also influence the protein stability of the HIF transcription factors. Intracellular oxygen level-sensing mechanisms rely on the activity of the proline-hydroxylases (PHDs) and the asparaginyl-hydroxylase activity of factor-inhibiting HIF (FIH). During normoxic conditions, these hydroxylases post-translationally mark the HIF-α subunits for proteasomal degradation, and in doing so, they prevent the HIF transcriptional activity [79,80,81,82,83,84]. Interestingly, the HIF-β subunits are stable under these conditions [79,80,81,82,83], thus indicating that the HIF regulation of the activity only occurs through the degradation of the alpha subunits. Hypoxia leads to the impairment of the PHDs and FIH activity and thus, an accumulation of the functional α-β-subunit HIFs complexes [79,80,81,82,83] that are responsible for the extensive transcriptional reprograming of cellular functions that allow the cells to survive and adapt to this stress response. HIFs, through a direct interaction with the hypoxia response elements (HREs) consensus sequences in their target genes, modulate their levels in order to switch their metabolism to that of the less energy efficient non-oxidative mitochondrial activity [9,85,86,87,88,89].

One of the important functions of HIFs is to prevent the conversion of pyruvate to acetyl-Co-A and to increase the expression of glucose transporters and glycolytic enzymes to emphasize the glycolytic pathway [90,91,92,93]. The HIFs also down-regulate cytochrome c oxidase (COX) subunit composition expression [94,95], and this is accompanied by a HIF-elevated expression of carbonic anhydrase 9 (*CA*-*IX*) and monocarboxylate transporter 4 (*MCT4*) that prevent the acidosis as a consequence of glycolysis-related proton release [96,97]. These glycolytic changes result in a lower ATP production, and the cellular energy requirements are limited by a HIF-mediated selective translational blockage [98,99,100]. This also promotes the induction of autophagy and mitophagy [100,101,102,103] by an-mTOR independent pathway [31,104].

**Figure 1:**
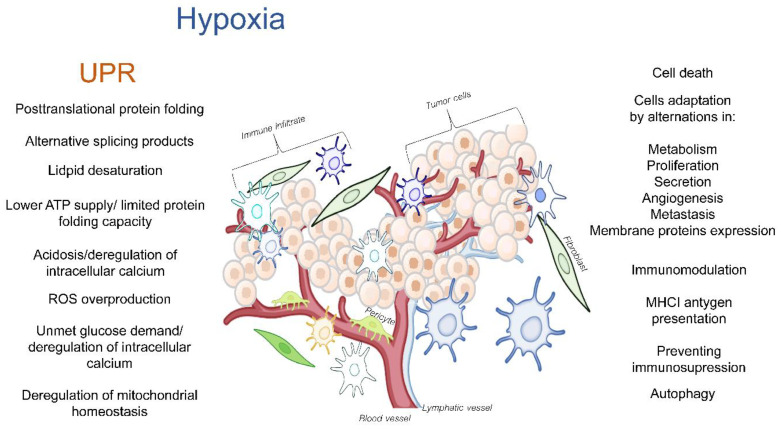
The hypoxia-related deregulation of ER homeostasis in TME cells that can result in activation of the UPR and UPRmt and subsequently modulate TME.

During this glycolytic switch, the cellular ATP-dependent processes such as protein synthesis, disulfide-bonds formation, peptide folding, and the maintenance of the redox potential and ion homeostasis are limited [49,105]. Furthermore, the hypoxia-related metabolic switch increases the production of lactic acid, thereby resulting in acidosis, the deregulation of intracellular calcium levels, and the overproduction of reactive oxygen species (ROS) [106,107,108]. Limited the glucose and glutamine demands would reduce the synthesis of uridine diphosphate-*N*-acetylglucosamine (UDP-GlcNAc) and thereby, limit the N-linked glycosylation in ER [109] as well as deregulate the ER calcium influx [110]. Furthermore, the changes in mitochondrial activity would result in the intracellular accumulation of ROS [111] since high amounts of ROS are generated as a byproduct of fatty acid β-oxidation [112,113,114]. Some of the TME deregulated cytokines and growth factors have also been reported to activate the NADPH oxidases and contribute to the ROS accumulation [45]. Finally, a prolonged hypoxia disturbs the protein import processes in the mitochondria as well as mitochondrial protein folding, and this activates the mitochondrial unfolded protein response (UPRmt) [115,116,117,118]. Hence, hypoxia, along with all these other factors, can deregulate the cellular proteostasis and consequently activate the UPR. This results in a complex molecular network of interactions that affect the TME, and consequently, the tumor’s progression (Figure 1).

Although HIF complexes containing HIF-1α subunits are considered to be the principal mediators of the cellular responses to hypoxia, in specific tissues, their functions can be supported and extended by the complexes that are formed by other α isoforms that include HIF-2α and HIF-3α [9,14,119,120,121,122,123,124]. The HIF-dependent transcriptional reprograming is not limited to a metabolic switch and facilitating cellular survival, but also to restoring oxygen homeostasis through promoting angiogenesis [9,13,125]. The HIFs are also responsible for increasing the levels of the vascular endothelial growth factor (*VEGF*) [11,126], matrix metalloproteinases (*MMP*) *2* and *13* [127], angiopoietin 2 (*ANGPT2*) [128], platelet-derived growth factor B (*PDGFB*) [129], placental growth factor (*PGF*) [130], and stem cell factor (*SCF*) [131] as well as endothelial nitric oxide synthase (*NOS3*) [123,132]. Furthermore, HIFs stimulate erythropoiesis [133,134,135] and help to maintain the necessary iron levels [136,137].

Hypoxia is only one of numerous processes that can induce an ER stress, and other ways include a nutrient deprivation, acidosis, a high metabolic demand, the processes of reactive oxygen species, an augmented secretory capacity, the deregulation of transcription and translation, and therapies that are related to the impact of cytotoxic drugs and radiation [45]. Depending on the specific pathological conditions of the TME, the involvement of UPR activation and the magnitude of this response may differ significantly. For example, the TME cells can utilize these signaling pathways for adaption and survival or they can lead to autophagy or apoptosis.

## 3. The UPR and UPRmt

The occurrence of an ER stress increases the demand for chaperones in the lumen of this organelle, and this leads to UPR initiation which begins with the glucose-regulated protein 78 (GRP78 also known as BiP (binding immunoglobin protein)) release from the luminal domains of three ER transmembrane sensors: protein kinase RNA-like endoplasmic reticulum kinase (PERK), inositol-requiring enzyme 1α (IRE1α), and activating transcription factor 6 (ATF6) [37,42]. BiP dissociation activates PERK and IRE1α via multimerization and trans-autophosphorylation, and this allows the ATF6 proteolytic maturation into an active ATF6f (p50) transcription factor to occur [138,139,140]. Upon their activation, all of these proteins initiate signaling pathways that function to help the cells to adapt to this insult, to repair the damage, and to restore ER homeostasis [141]. The ATF6f promotes the synthesis of the protein chaperones (including BiP) and the ER membrane lipids, the ER-associated degradation (EDEM) of the misfolded proteins, and it enhances N-glycosylation [142,143]. IRE1α is also responsible for IRE1-dependent decay (RIDD) that degrades selected mRNAs in order to reduce the ER load [144,145,146,147] as well as IRE1 α splices the mRNA transcript of X-box binding-protein (XBP) transcription factor into its transcriptionally active isoform (*XBP1s*) [148]. XBP1s promote the ER membrane’s biosynthesis and support its folding capacity [9,36,148,149]. The main consequence of PERK activation is the phosphorylation of the alpha subunit of the eukaryotic initiation factor eIF2. This promotes a general suppression of protein synthesis [42,150,151], and it allows for the increased expression of specific proteins including (1) growth arrest and DNA damage inducible protein (GADD34), (2) proapoptotic CCAAT/enhancer binding homologous protein (CHOP), and (3) activating transcription factor 4 (ATF4). ATF4 transcriptionally supports the adaptation to an ER stress and protein folding [152], whereas GADD34 enables the dephosphorylation of eIF2, and thus removes the translational blockage when the ER stress is mitigated [153].

In non-malignant cells, if the UPR stress response is too persistent or too intense, the cell death pathways are initiated. Although the accumulation of ATF4 and the PERK-dependent proapoptotic factor CHOP are well recognized as cell death signals, IRE1 can stimulate the Janus N-terminal kinase (JNK) to increase the expression of the death receptor 5 (*DR5*), and ATF6f can also contribute to the CHOP accumulation [9,144,154,155]. The UPR is also accompanied by complex changes in many other apoptotic proteins such as the p53 upregulated modulator of apoptosis (PUMA), phorbol-12-myristate-13-acetate-induced protein 1 (PMAIP1, also known as NOXA), and growth arrest and DNA damage -inducible alpha GADD45A [141,146,156,157,158,159], which together with the main signals, influence the cell’s fate. Furthermore, the UPR-specific roles of noncoding RNAs also play a role. For example, all of the UPR pathways modulate specific miRNA levels in order to prevent to extensive accumulation of proadaptive or apoptotic proteins [9,44,160,161,162,163]. Although the intrinsic apoptotic pathway is the main mechanism that is responsible for cell death during the UPR, recent studies indicate that the PERK branch of UPR can lead to autophagy [164,165] and necroptosis [166,167,168,169,170,171]. The activation of the latter pathways in hypoxic conditions prevents the HIF-dependent metabolic switch and thus, this increases the intracellular ROS accumulation [172,173,174,175].

In the TME cancer cells, the UPR-specific signals are often clouded via the oncogenic transformations [45] given that activation of the oncogenes results in an increased protein and membrane synthesis [176]. Furthermore, the cancer cells adapt to avoid the UPR cell death signals as illustrated by the MYC Proto-Oncogene example. Increased levels of this oncogene in normal cells results in apoptosis, whereas in cancers cells, both the XBP1s and IRE1 allow for the cells to avoid an MYC-related cell death [177,178,179]. The IRE1 pathway also modulates some of the effects of the mutant RAS, however, the significance of this for cancer cell survival remains unclear [180].

Finally, the hypoxia-related metabolic switch and the resulting energetic deficiency may disrupt the mitochondrial protein homeostasis and lead to the activation of the mitochondrial UPR (UPRmt). This could occur via the limiting of the influx of the nuclear-encoded proteins and interfere with mitochondrial refolding after the protein import and activation of the UPRmt [115,116,117,118,181]. The UPRmt is a proadaptive mechanism that changes the expression of both the mitochondrial and nuclear encoded genes (including *ATF5* and *ATF4*) to restore homeostasis or if this fails, it leads to apoptosis [115,116,117,118]. Notably, the UPRmt-related activation of the PERK pathway has also been reported [115,116,117,118,182].

## 4. The Crosstalk between Hypoxia and UPR in the TME

Although the pronounced activation of UPR signaling in cancer has been reported mainly for extreme hypoxic conditions [65,146], numerous reports have indicated that particular aspects of this are triggered even in less oxygen-limiting conditions, including increased BIP expression [33,123,183,184,185,186,187] and PERK-related activity [73,184,188,189,190,191,192]. The hypoxia-elevated BiP levels result from the activities of the extracellular signal-regulated kinase (ERK) and protein kinase C (PKC) [184]. Furthermore, the increase in BIP expression can result from the hypoxic induction of its cofactor and transcriptional inducer called the cell migration-inducing and hyaluronan-binding ER protein (CEMIP) [193]. In the ER, CEMIP/GRP78 support the adaptive responses by raising the intracellular calcium levels and subsequently increasing the PKCα activity [193].

Although the PERK-dependent translational attenuation occurs immediately during acute hypoxia, this blockage is removed after a prolonged hypoxic exposure or with increased oxygen levels [188,194,195]. The PERK-related eIF2 phosphorylation is also present in the transient (cyclic hypoxia) models [196,197,198,199,200], and thus, the activation of this branch of UPR is very plausible in solid tumors that are characterized by fluctuating oxygen concentrations during tumor expansion. PERK activation inhibits the HIF-1α translation in cancer cells and thus, limits the HIF-1 transcriptional activity [201].

The transcriptional activity of PERK-preferentially translated ATF4 is limited by PHD1, whereas ATF4 was shown to destabilize PHD3 and thus, support the HIF-1α accumulation [202]. The question of whether this mechanism serves as a buffer of the HIF-1-related cellular adaptation or contributes to cell death will require further research.

Interestingly, the PERK inhibition in hypoxia-exposed cells promotes accelerated cell death [188]. In agreement with these findings, other studies have shown that in several cancer cell lines, PERK signaling is required to stimulate the autophagosome formation through the stimulating transcription of the autophagy genes microtubule-associated protein 1 light chain 3 beta (*MAP1LC3B*) [46,74,141]. Furthermore, the PERK activity induces carbonic anhydrase 9 (CA9) and thus, it prevents hypoxia-induced acidosis [106,203], whereas ATF4 activity reduces the hypoxia-related damage and supports maintaining a redox balance and mitochondrial homeostasis [115,116,117,118,182]. Notably, increased ATF4 expression is found in many hypoxic and nutrient-deprived tumors [204], and it has been shown to mediate autophagy under hypoxia [74,205]. PERK and ATF4 have a protective role in oxidative damage in glioblastoma cells that are exposed to cyclic hypoxia or radiotherapy [197,206]. In human cervical cancer, PERK activation during hypoxia results in the accumulation of oncogenic lysosomal-associated membrane protein 3 (LAMP3) and in the increased aggressiveness of these cells [197].

Although PERK activation during hypoxia can lead to increased levels of CHOP expression and cell death in normal cells [179,207,208,209], in tumors, the CHOP expression is not elevated as dramatically as it is in the pharmacologically induced ER stress [188]. Furthermore, CHOP can also serve in a proadaptive role by limiting the activity of the endothelial nitric synthase (*NOS3, eNOS*) [210] and preventing the ROS accumulation through the hypoxic uncoupling of this enzyme [44,211,212].

Despite the fact that the PERK pathway has been considered as the main response pathway of the UPR in hypoxic tumors, the activation of the two other branches occurs as well. The elevated expression of ATF6-dependent prosurvival genes in response to hypoxia has been reported in gastric tumors [213] and mutant p53 cancer cells [214]. Furthermore, the elevated levels of these transcription factors are a hallmark of a poor prognosis for pancreatic cancer patients [16]. The hypoxic activation of ATF6 signaling in the TME, however, has not been convincingly presented so far [188].

Although elevated levels of XBP1s have been reported in many types of cancers and this has been correlated with poor prognosis, the IRE1 activity and the related accumulation of XBP1s has been reported mainly in cells that have been exposed acute and moderate hypoxia [146,188,193,215,216,217,218,219,220,221]. However, acute hypoxia can inhibit IRE1 and lead to reduced XBP1s levels [222]. Studies in endothelial cells have indicated that the IRE1 activity was necessary for maintaining the proper HIF-1α expression that was independent of the XBP1s [223]. This finding is supported by reports from other endothelial cell studies, where despite the apparent hypoxia-related IRE1 activity, the XBP1s’ induction was not observed [224]. Although, this suggests that the hypoxic activation of IRE1 may serve a different role than it does during the canonical UPR, it still modulates the adaptive response to hypoxia. Further analyses will be required to decipher the significance of these findings [75,225,226,227,228]. It is also plausible that the involvement and significance of the IRE1/XBP1s axis is cancer type-specific given that in breast cancer, the XBP1s were shown to interact with HIF-1α to cooperatively reprogram the cellular expression including the expression of glucose transporter 1 (*GLUT1*) and lactate dehydrogenase A (*LDHA*) [146,193,218]. In contrast, in colon cancer, this interaction was prevented by the hyper-activated WNT/β-catenin axis to limit the HIF-1 activity [193]. Similar mechanisms were observed in breast cancer cells during acute hypoxia, however, during prolonged hypoxia, the XBP1s induced an miR-153 expression, and this is a negative regulator of HIF-1α [215,216].

Finally, an elegant HIF-dependent mechanism that could result in a complete UPR activation has been proposed in endothelial cells where HIF-1 induces *VEGF* through the stimulation of its receptors (VEGFRs), it activates phospholipase C (PLC), and thus, this leads to a phosphate (IP3)-dependent calcium release that initiates the UPR [229,230]. Furthermore, ATF6f, XBP1s, and ATF4 increase the expression of the proangiogenic genes including *VEGF,* and this suggests that the UPR supports hypoxia-related angiogenesis [231,232,233,234,235,236,237,238,239,240,241,242]. In contrast, the PERK/ATF4 signals are limiting factors for erythropoietin (EPO) expression [58].

Numerous reports have indicated that HIFs are very effective in preventing ROS formation during chronic hypoxia [92,95,243,244,245,246], and this may prevent the full activation of both the UPR and the UPRmt. Importantly, however, intermittent hypoxia (that is termed as chronic exposure of cells to cycles of hypoxia/reoxygenation) accompanies the development of the majority of solid tumors, which were also subject to persistent UPR activation [247,248]. One of the important consequences of temporal normoxia in hypoxic cells is the extensive ROS accumulation upon the reintroduction of oxygen that is also observed during hypoxia-reoxygenation injury and ischemia-reperfusion injury. This results in extensive UPR and UPRmt activation [95,177,243,249,250,251,252,253,254,255,256,257,258,259,260,261,262,263,264,265,266,267,268,269,270,271,272,273,274]. Unfortunately, however, the vast majority of hypoxia-related cancer research utilizes continuous exposure to low oxygen levels, and this may underestimate the level that is needed for the UPR activation.

PERK and IRE1, through their inhibitory effects on HIF-1α stability and transcriptional activity, could contribute the transition from HIF-1 to HIF-2 signaling in both endothelial and cancer cells that is observed during prolonged hypoxia, and this allows for a better adaptation to this insult [9,70,88,89]. Importantly, the HIF-mediated cellular adaptation to hypoxia relies on the induction of angiogenesis. This requires the elevated secretion of the proangiogenic factors and the increased expression of their specific transmembrane receptors as well as the remodeling of the extracellular space thorough the secreted enzymes. On one hand, all of these proteins modulate the TME, whereas on the other, these proteins need to mature properly, which requires that the ER homeostasis must be preserved [35,76,275,276,277,278]. Taken together, the crosstalk between HIF signaling and UPR clearly demonstrates that the interaction between these pathways is dynamic. Furthermore, the final proadaptive or proapoptotic consequences result from the multiple networks of the temporal transcriptional and posttranscriptional signals originating from both of these pathways. The hypoxia-related involvement of the UPR is strongly oxygen concentration-dependent, and therefore, the crosstalks between these pathways in the TME will differ within the different tumor regions. Hence, cell location and kinetic approaches are needed to better understand the molecular mechanisms that are connecting the hypoxic signaling with the UPR.

The hypoxia-induced activation of the UPR can also facilitate metastasis and dormancy [279,280]. Both HIF-1 and XBP1s are reported to upregulate lysyl oxidase (LOX) in the estrogen receptor-negative breast tumors and thus promote a pre-metastatic niche formation [106,281]. Furthermore, active PERK is an important prosurvival pathway in cells that are undergoing an epithelial-to-mesenchymal transition (EMT) with an enhanced secretory capacity [282].

**Figure 2:**
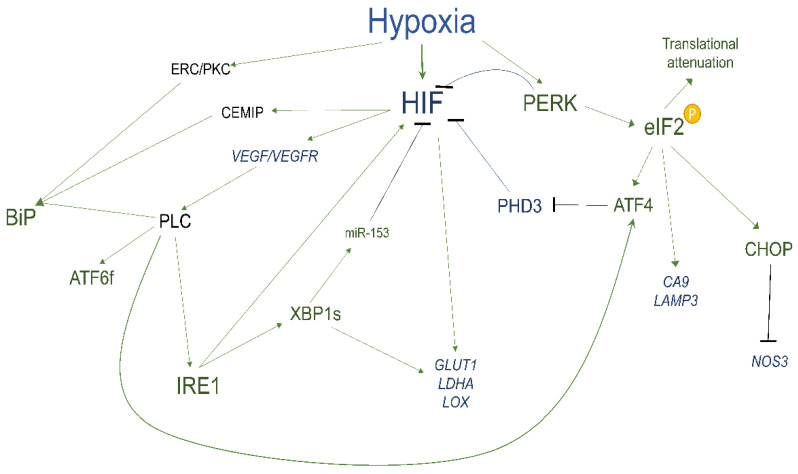
The crosstalk between UPR and hypoxia signaling. During hypoxia, the accumulation of misfolded/unfolded proteins in ER and mitochondria activate PERK signaling, and this contributes to both pro-survival (global translational arrest and induction of pro-angiogenic genes) and apoptotic responses (induction of *CHOP* and inhibition of pro-angiogenic *eNOS* expression). Furthermore, in some models, the hypoxia-related activation of ATF6 and IRE1α contribute to pro-survival and pro-angiogenic signaling. There also appears to be cooperation between XBP1s and HIF1 in pro-survival signaling.

The hypoxia-induced UPR in cancer cells can also affect the function of the immune component of the TME and thus, limit the therapeutic approaches [45]. Both XBP1s and ATF6 reduce the surface expression of major histocompatibility complex class I (MHC-I) molecules [283], whereas PERK translational inhibition impairs their ability to present peptides to MHC-1 molecules [62]. We have demonstrated that XBP1-induced miR-346 reduces the peptides influx to the ER and MHC-I assembly [36]. All of these findings suggest that the UPR can help cancer cells to attenuate CD8^+^ T cell responses and thus, limit the efficiency of the immunotherapeutic approaches. Furthermore, numerous studies have indicated that cancer cells with an active UPR can drastically alter the function of the immune cells including natural killer cells, macrophages, T-cells, and myeloid cells in the TME mainly through the IRE1-XBP1s driven expression of the proinflammatory factors as well as the PERK signals. Depending on the cancer model, however, the UPR activation can support or prevent the antitumor immune responses [269,284,285,286,287,288,289,290,291,292,293,294,295], and therefore further studies are required to better understand the UPR effects on the immune recognition in the TME in different cancer types. The complex network of molecular interaction between hypoxia and UPR is summarized in Figure 2 and Table 1.

Notably, although the hypoxia-related activation of the UPR in intratumoral immune cells has not been convincingly reported, the ROS accumulation and acidosis in the TME are known ER stressors of tumor-infiltrating leukocytes [45]. The ROS accumulation during cyclic hypoxia in solid tumors is probably underestimated, and the level of activation of the UPR in the intratumoral immune cells is unclear and therefore, further research is required to understand this process.

## 5. UPR Activation in TME Cells Depends on Hypoxia Dynamics and Severity

The tumors are extremely heterogenous in terms of their microregions of oxygenation as well as the severity of the hypoxia that ranges from moderate oxygen deprivation to anoxia [72,296,297]. Furthermore, oxygen availably in the TME is often highly dynamic, and it is characterized by the periodic cycling of cells between various levels of oxygenation. Hence, depending on the nature of hypoxia, the cells are exposed in the TME to different levels of UPR activation as summarized below.

### 5.1. Anoxia and Extreme Acute Hypoxia

The tumor vasculature is often immature and lacks smooth muscle cells that along with it having high interstitial pressures, can result in drastic perfusion changes, including the temporary shutdown of vessels. This results in acute hypoxia or even anoxia of some small tumor regions (oxygen 0–0.1%) [298,299]. Notably, the tumor cells are able to survive in anoxic conditions for prolonged periods of time [74]. Such conditions result in the complete activation of UPR signaling [65,146]. This includes an increase in BiP expression that is accompanied by the rapid PERK activation and translational inhibition [188,300], and IRE1-mediated XBP1 splicing [146,215,217,218,219,220,221]. However, during the chronic exposure to acute hypoxia (above 4h), the eIF2α phosphorylation levels are a partially restored [188,300].

### 5.2. Moderate and Mild Hypoxia

The outpacing of a new blood supply compared to the rate of the tumor’s growth and the abnormal architecture of the newly formed blood vessels often have less dramatic consequences on the oxygen delivery to the tumor regions [301,302]. Consequently, for many of the TME cells, their oxygen availability is higher, and these cells are exposed to moderate (0.1–1% oxygen) or mild hypoxia (form 1–3%). Under these conditions, the activation of the UPR requires a longer time for it to occur, and it occurs mainly during periods of chronic exposure to hypoxia. For example, the phosphorylation of eIF2α requires more than 8 h if the oxygen concentration is moderate and consequently, a translational blockage is accompanied by the mTOR inhibition [70]. Furthermore, a recent study has shown that during chronic mild hypoxia, the ER stress attenuates HIF-1 and HIF-2 signaling by promoting the degradation of their alpha subunits independent of the von Hippel-Lindau (VHL) pathway. In this case, the UPR is activated by the glycogen synthase kinase-3 beta (GSK3β) and the ubiquitin ligase FBXW1A/βTrCP [303].

### 5.3. Intermittent Hypoxia

Importantly, the transient changes in the blood flow, which are independent of the overall tumor oxygenation status result in large fluctuations in the tumor pO_2_ levels that temporally increase the hypoxia severity. Such fluctuations usually can occur due to transient changes in the blood flow, and they are independent of the treatment or the overall tumor oxygenation status. As the blood flow changes from high to low, the proportion and severity of the hypoxia increases. Hence, the cells are exposed to continuous cycles of severe hypoxia, which is followed by reoxygenation. Additionally, these fluctuations can last from 30 min to 2 h [304,305]. Despite these relatively short period of hypoxia, this time is sufficient to switch the metabolism to the glycolytic pathway [200,306]. However, the restored oxygen availability will lead to the accumulation of ROS due to inability of the mitochondria to rapidly utilize “an extra” oxygen. This will then be accompanied by the rapid HIF-α degradation due to the reactivation of the PHDs, and the impairment of the HIF-related protection from the oxidative stress [307,308]. Hence, intermittent severe and moderate hypoxia lead to the extensive and persistent activation of the UPR and the UPRmt pathways [95,177,243,247,248,249,250,251,252,253,254,255,256,257,258,259,260,261,262,263,264,265,266,267,268,269,270,271,272,273,274].

## 6. Conclusions

Although our current understanding of the molecular crosstalk between the UPR and hypoxia in the tumor microenvironment remains fairly limited, and presently, it is beyond any sort of therapeutic control, understanding these interactions could potentially change that. Despite our understanding of these stress pathways that has been presented in some detail in both malignant and non-malignant cells, the complexity of the activated signaling pathways remain a research barrier. A major problem is the constant variability of the different cancers as well as the complexity of the TME which often lead to apparently contradictory results. The extent of the hypoxia-related ER stress and the course of the UPR can drastically differ depending on the oxygen availability, the time of the exposure to hypoxia, as well as the type of oncogenic transformation. Furthermore, the majority of current studies often utilize two-dimensional in vitro cultures of cancer cells that are exposed to continuous hypoxia and this approach may not mimic the dynamic and cyclic conditions that are found in solid tumors.

Despite the fact that hypoxia is only one of several inducers of the UPR in the TME, solid tumors are exposed to intermittent hypoxia that results in the accumulation of high ROS levels and the complete activation of the UPR [309,310,311,312,313,314]. Furthermore, depending on the tissue, the cells often differ in their physiological oxygen needs, and consequently in their sensitivity to reoxygenation [315]. Hence, now we are witnessing the rapid development of organoid and 3-D culture models as well as single cell sequencing techniques [316,317]. Furthermore, the new approaches should evolve into more complex and relevant model systems containing both a fluctuating oxygen level component as well as the complex 3-D structure of the tumor environment.

Many of the current research models focus on understanding the hypoxia and the ER stress signaling based on steady state models utilizing a single arbitrary time of exposure. This approach does not take into account that the signaling pathways are dynamic and often changing. Furthermore, although HIFs, PERK, and IRE1 are crucial regulators of these cellular responses, their activities are accompanied by the genome-wide reprograming of the transcriptional and translational pathways. Hence, the studies focusing on analyzing the time-related profiles of the development of these signaling pathways, especially in a genome-wide context, will dramatically enhance our understanding of the crosstalk between these complex pathways. Although such approaches are very challenging, the development and increased availability of the next generation sequencing techniques, including single cell sequencing [318,319], should help to overcome these above-mentioned limitations.

A large fraction of the hypoxia-related research is solely based on use of hypoxia mimetics that function by preventing HIF-α subunit degradation in order to activate HIF signaling [320,321]. However, these compounds provide only a limited insight into the complexity of the changes in the cellular transcriptome, proteome, and metabolism that occur during hypoxia. Clearly, results that have been obtained in these models need be verified in limited oxygen conditions. Similarly, although ER homeostasis can be disturbed by many pharmacological ER stressors, depending on their mechanism of action, dosage, and time of exposure, the course of the UPR and the cell’s fate will differ [141,163,322]. Taken together, although the chemical approaches to mimic the hypoxia-related activation of UPR can be used, their utility remains limited, and it should always require more physiological validations.

Nevertheless, the recent development of hypoxia-responsive nanoparticles that selectively release their cargo under hypoxic conditions in the TME [323,324] provides a perspective for the specific delivery of the UPR branches inhibitors including 4µ8C for IRE1 [325,326], ISRIB for PERK [327], or Ceapins for ATF6 [328]. This approach would provide an excellent mechanism for the selective analysis of these pathway functions in the context of the hypoxic TME.

Tumor angiogenesis relies on non-malignant endothelial cells that receive the signals from the cancer cells and are also subject to cellular insults such as hypoxia and UPR activation [46]. Furthermore, the oncogenic transformations of the malignant cells often blunt the meaning of the signaling pathways, and thus, more research in non-malignant cells is needed to obtain proper insight into both the UPR and the cellular response to hypoxia. Although the past decade has resulted in defining the complex, novel non-coding RNA based regulatory networks that modulate both hypoxia and the UPR [36,44,89,123,163,200,290,329,330,331,332,333,334,335], we are just to beginning to appreciate their role in modulating the TME.

Taken together, depending on the cancer type, the importance of both hypoxia and UPR signaling in the disease aggressiveness, progression, and therapy often differ. Some of these contradictions are the consequence of oncogenic transformations, whereas the others result from different experimental approaches that the researchers are using (oxygen concentration, time of hypoxic exposure, and cyclic hypoxia). Unfortunately, there is no good solution to this problem other than effectively developing more relevant cancer specific 3-D models and dynamic conditions in order to gain a better understanding of the means by which cancers avoid apoptosis or immune responses through their modulation of the UPR pathways.

## Figures and Tables

**Table 1:** The crosstalk between UPR and hypoxia signaling.

UPR	Hypoxia	Molecular Background	Ref.
General induction of UPR including BiP expression	Anoxia, acute extreme hypoxia, mild hypoxia (chronic), intermittent hypoxia	CEMIP induces BIP expression; ERK/PKC activates UPR; HIF-1 through VEGFRs and PLC activates UPR.	[65,146,193] [184] [229,230]
Activation of PERK signaling	Anoxia, acute hypoxia, moderate hypoxia (chronic), intermittent hypoxia	PERK activation inhibits HIF-1α translation; PERK induces *CA9* expression; ATF4 reduces hypoxia-related damage and supports maintaining a redox balance and mitochondrial homeostasis; ATF4 destabilizes PHD3 to support HIF-1α accumulation; CHOP limits *NOS3* activity; PERK/ATF4 limit *EPO* expression.	[201] [106,203] [115,116,117,118,182] [202] [210] [58]
Activation of IRE1 signaling	Anoxia, acute hypoxia, moderate hypoxia (chronic), intermittent hypoxia	Acute hypoxia inhibits IRE1 and reduce XBP1s levels; IRE1 activity is necessary for maintaining proper HIF-1α expression; During prolonged hypoxia, XBP1s induces miR-153 to reduce HIF-1α; XBP1s interacts with HIF-1α to cooperatively stimulate expression of *GLUT1* and *LDHA*.	[222] [223] [215,216] [146,193,217]
ATF6	Anoxia, acute hypoxia, moderate hypoxia (chronic), intermittent hypoxia	Along with ATF4 and XBP1 supports expression of *VEGF.*	[231,232,233,234,235,236,237,238,239,240,241,242]

## Data Availability

Not applicable.

## References

[1] Balkwill F.R., Capasso M., Hagemann T. (2012). The tumor microenvironment at a glance. J. Cell Sci..

[2] Petrova V., Annicchiarico-Petruzzelli M., Melino G., Amelio I. (2018). The hypoxic tumour microenvironment. Oncogenesis.

[3] Pouyssegur J., Dayan F., Mazure N.M. (2006). Hypoxia signalling in cancer and approaches to enforce tumour regression. Nature.

[4] Siemann D.W. (2011). The unique characteristics of tumor vasculature and preclinical evidence for its selective disruption by tumor-vascular disrupting agents. Cancer Treat. Rev..

[5] Strzyz P. (2016). Hypoxia as an off switch for gene expression. Nat. Rev. Mol. Cell. Biol..

[6] Thiele R.H. (2017). Subcellular energetics and metabolism: A cross-species framework. Anesth. Analg..

[7] Wheaton W.W., Chandel N.S. (2011). Hypoxia. 2. Hypoxia regulates cellular metabolism. Am. J. Physiol.-Cell Physiol..

[8] Bargiela D., Burr S.P., Chinnery P.F. (2018). Mitochondria and hypoxia: Metabolic crosstalk in cell-fate decisions. Trends Endocrinol. Metab. TEM.

[9] Bartoszewska S., Cabaj A., Dabrowski M., Collawn J.F., Bartoszewski R. (2019). *Mir-34c-5p* modulates x-box-binding protein 1 (xbp1) expression during the adaptive phase of the unfolded protein response. FASEB J..

[10] Choi K.S., Bae M.K., Jeong J.W., Moon H.E., Kim K.W. (2003). Hypoxia-induced angiogenesis during carcinogenesis. J. Biochem. Mol. Biol..

[11] Krock B.L., Skuli N., Simon M.C. (2011). Hypoxia-induced angiogenesis: Good and evil. Genes Cancer.

[12] LaManna J.C., Kuo N.T., Lust W.D. (1998). Hypoxia-induced brain angiogenesis. Signals and consequences. Adv. Exp. Med. Biol..

[13] Cabaj A., Moszynska A., Charzynska A., Bartoszewski R., Dabrowski M. (2022). Functional and hre motifs count analysis of induction of selected hypoxia-responsive genes by hif-1 and hif-2 in human umbilical endothelial cells. Cell. Signal..

[14] Jaskiewicz M., Moszynska A., Serocki M., Kroliczewski J., Bartoszewska S., Collawn J.F., Bartoszewski R. (2022). Hypoxia-inducible factor (hif)-3a2 serves as an endothelial cell fate executor during chronic hypoxia. EXCLI J..

[15] Qiu G.Z., Jin M.Z., Dai J.X., Sun W., Feng J.H., Jin W.L. (2017). Reprogramming of the tumor in the hypoxic niche: The emerging concept and associated therapeutic strategies. Trends Pharmacol. Sci..

[16] Xiao W., Cao R.C., Yang W.J., Tan J.H., Liu R.Q., Kan H.P., Zhou L., Zhang N., Chen Z.Y., Chen X.M. (2022). Roles and clinical significances of atf6, emc6, and apaf1 in prognosis of pancreatic cancer. Front. Genet..

[17] Boreel D.F., Span P., Peters H., van den Bijgaart R.J.E., Heskamp S., Adema G.J., Bussink J. (2021). Targeting oxygen metabolism reduces hypoxia in the tumor microenvironment of a syngeneic mouse model. Mol. Cancer Ther..

[18] Kheshtchin N., Hadjati J. (2022). Targeting hypoxia and hypoxia-inducible factor-1 in the tumor microenvironment for optimal cancer immunotherapy. J. Cell. Physiol..

[19] Sun X.L., Luo H., Han C.B., Zhang Y., Yan C.L. (2021). Identification of a hypoxia-related molecular classification and hypoxic tumor microenvironment signature for predicting the prognosis of patients with triple-negative breast cancer. Front. Oncol..

[20] Liu Z., Tang Q., Qi T.Z., Othmane B., Yang Z., Chen J.B., Hu J., Zu X.B. (2021). A robust hypoxia risk score predicts the clinical outcomes and tumor microenvironment immune characters in bladder cancer. Front. Immunol..

[21] Arvindam U.S., Kennedy P., Ettestad B., Phung S.K., Hinderlie P., Lim J., Miller J.S., Felices M. (2021). Hypoxia profoundly alters natural killer cell phenotype and function: Implications for immunotherapy within the solid tumor microenvironment. J. Immunol..

[22] Gastelum G., Veena M., Lyons K., Lamb C., Jacobs N., Yamada A., Baibussinov A., Sarafyan M., Shamis R., Kraut J. (2021). Can targeting hypoxia-mediated acidification of the bone marrow microenvironment kill myeloma tumor cells?. Front. Oncol..

[23] He X.B., Ding J.F., Cheng X., Xiong M.M. (2021). Hypoxia-related gene-based signature can evaluate the tumor immune microenvironment and predict the prognosis of colon adenocarcinoma patients. Int. J. Gen. Med..

[24] Pei J.P., Zhang C.D., Yusupu M., Zhang C., Dai D.Q. (2021). Screening and validation of the hypoxia-related signature of evaluating tumor immune microenvironment and predicting prognosis in gastric cancer. Front. Immunol..

[25] Jiang F., Miao X.L., Zhang X.T., Yan F., Mao Y., Wu C.Y., Zhou G.P. (2021). A hypoxia gene-based signature to predict the survival and affect the tumor immune microenvironment of osteosarcoma in children. J. Immunol. Res..

[26] Augustin R.C., Delgoffe G.M., Najjar Y.G. (2020). Characteristics of the tumor microenvironment that influence immune cell functions: Hypoxia, oxidative stress, metabolic alterations. Cancers.

[27] Krishnamachary B., Mironchik Y., Jacob D., Goggins E., Kakkad S., Ofori F., Dore-Savard L., Bharti S.K., Wildes F., Penet M.F. (2020). Hypoxia theranostics of a human prostate cancer xenograft and the resulting effects on the tumor microenvironment. Neoplasia.

[28] Gray M., Meehan J., Turnbull A.K., Martinez-Perez C., Kay C., Pang L.Y., Argyle D.J. (2020). The importance of the tumor microenvironment and hypoxia in delivering a precision medicine approach to veterinary oncology. Front. Vet Sci..

[29] Vito A., El-Sayes N., Mossman K. (2020). Hypoxia-driven immune escape in the tumor microenvironment. Cells.

[30] Bensellam M., Maxwell E.L., Chan J.Y., Luzuriaga J., West P.K., Jonas J.C., Gunton J.E., Laybutt D.R. (2016). Hypoxia reduces er-to-golgi protein trafficking and increases cell death by inhibiting the adaptive unfolded protein response in mouse beta cells. Diabetologia.

[31] Wouters B.G., Koritzinsky M. (2008). Hypoxia signalling through mtor and the unfolded protein response in cancer. Nat. Rev. Cancer.

[32] Maamoun H., Benameur T., Pintus G., Munusamy S., Agouni A. (2019). Crosstalk between oxidative stress and endoplasmic reticulum (er) stress in endothelial dysfunction and aberrant angiogenesis associated with diabetes: A focus on the protective roles of heme oxygenase (ho)-1. Front. Physiol..

[33] Binet F., Sapieha P. (2015). Er stress and angiogenesis. Cell Metab..

[34] Martinez J.A., Banerjee D.K. (2000). Tunicamycin inhibits angiogenesis by er stress. Glycobiology.

[35] Bartoszewski R., Kroliczewski J., Piotrowski A., Jasiecka A.J., Bartoszewska S., Vecchio-Pagan B., Fu L., Sobolewska A., Matalon S., Cutting G.R. (2016). Codon bias and the folding dynamics of the cystic fibrosis transmembrane conductance regulator. Cell. Mol. Biol. Lett..

[36] Bartoszewski R., Brewer J.W., Rab A., Crossman D.K., Bartoszewska S., Kapoor N., Fuller C., Collawn J.F., Bebok Z. (2011). The unfolded protein response (upr)-activated transcription factor x-box-binding protein 1 (xbp1) induces microrna-346 expression that targets the human antigen peptide transporter 1 (tap1) mrna and governs immune regulatory genes. J. Biol. Chem..

[37] Almanza A., Carlesso A., Chintha C., Creedican S., Doultsinos D., Leuzzi B., Luis A., McCarthy N., Montibeller L., More S. (2019). Endoplasmic reticulum stress signalling—From basic mechanisms to clinical applications. FEBS J..

[38] Walter P., Ron D. (2011). The unfolded protein response: From stress pathway to homeostatic regulation. Science.

[39] Bravo R., Parra V., Gatica D., Rodriguez A.E., Torrealba N., Paredes F., Wang Z.V., Zorzano A., Hill J.A., Jaimovich E. (2013). Endoplasmic reticulum and the unfolded protein response: Dynamics and metabolic integration. Int. Rev. Cell Mol. Biol..

[40] Karagoz G.E., Acosta-Alvear D., Walter P. (2019). The unfolded protein response: Detecting and responding to fluctuations in the protein-folding capacity of the endoplasmic reticulum. Cold Spring Harb. Perspect. Biol..

[41] Petrillo S., Chiabrando D., Genova T., Fiorito V., Ingoglia G., Vinchi F., Mussano F., Carossa S., Silengo L., Altruda F. (2018). Heme accumulation in endothelial cells impairs angiogenesis by triggering paraptosis. Cell Death Differ..

[42] Hetz C. (2012). The unfolded protein response: Controlling cell fate decisions under er stress and beyond. Nat. Rev. Mol. Cell. Biol..

[43] Kim R., Emi M., Tanabe K., Murakami S. (2006). Role of the unfolded protein response in cell death. Apoptosis.

[44] Gebert M., Bartoszewska S., Janaszak-Jasiecka A., Moszynska A., Cabaj A., Kroliczewski J., Madanecki P., Ochocka R.J., Crossman D.K., Collawn J.F. (2018). Piwi proteins contribute to apoptosis during the upr in human airway epithelial cells. Sci. Rep..

[45] Chen X., Cubillos-Ruiz J.R. (2021). Endoplasmic reticulum stress signals in the tumour and its microenvironment. Nat. Rev. Cancer.

[46] Bartoszewska S., Collawn J.F. (2020). Unfolded protein response (upr) integrated signaling networks determine cell fate during hypoxia. Cell. Mol. Biol. Lett..

[47] Ron D. (2011). Protein folding homeostasis in the endoplasmic reticulum. FEBS J..

[48] Naidoo N. (2011). Protein folding in the endoplasmic reticulum. Comprehensive Biotechnology: Scientific Fundamentals of Biotechnology.

[49] Braakman I., Bulleid N.J. (2011). Protein folding and modification in the mammalian endoplasmic reticulum. Annu. Rev. Biochem..

[50] Kleizen B., Braakman I. (2004). Protein folding and quality control in the endoplasmic reticulum. Curr. Opin. Cell Biol..

[51] Helenius A., Marquardt T., Braakman I. (1992). The endoplasmic reticulum as a protein-folding compartment. Trends Cell Biol..

[52] Hagiwara M., Nagata K. (2012). Redox-dependent protein quality control in the endoplasmic reticulum: Folding to degradation. Antioxid Redox Sign.

[53] Braakman I. (2005). Disulfide bond formation during protein folding in the endoplasmic reticulum. FEBS J..

[54] Braakman I., Hebert D.N. (2013). Protein folding in the endoplasmic reticulum. Cold Spring Harb. Perspect. Biol..

[55] Bartoszewski R., Rab A., Twitty G., Stevenson L., Fortenberry J., Piotrowski A., Dumanski J.P., Bebok Z. (2008). The mechanism of cystic fibrosis transmembrane conductance regulator transcriptional repression during the unfolded protein response. J. Biol. Chem..

[56] Heindryckx F., Binet F., Ponticos M., Rombouts K., Lau J., Kreuger J., Gerwins P. (2016). Endoplasmic reticulum stress enhances fibrosis through ire1 alpha-mediated degradation of mir-150 and xbp-1 splicing. EMBO Mol. Med..

[57] Karamanos N.K., Theocharis A.D., Piperigkou Z., Manou D., Passi A., Skandalis S.S., Vynios D.H., Orian-Rousseau V., Ricard-Blum S., Schmelzer C.E.H. (2021). A guide to the composition and functions of the extracellular matrix. FEBS J..

[58] Chiang C.K., Nangaku M., Tanaka T., Iwawaki T., Inagi R. (2013). Endoplasmic reticulum stress signal impairs erythropoietin production: A role for atf4. Am. J. Physiol.-Cell Physiol..

[59] Manalo R.V.M. (2017). Anastasis and the er stress response: Solving the paradox of the unfolded protein response in cancer. Med. Hypotheses.

[60] Vandewynckel Y.P., Laukens D., Geerts A., Bogaerts E., Paridaens A., Verhelst X., Janssens S., Heindryckx F., Van Vlierberghe H. (2013). The paradox of the unfolded protein response in cancer. Anticancer Res..

[61] Hewitt E.W. (2003). The mhc class i antigen presentation pathway: Strategies for viral immune evasion. Immunology.

[62] Granados D.P., Tanguay P.L., Hardy M.P., Caron E., de Verteuil D., Meloche S., Perreault C. (2009). Er stress affects processing of mhc class i-associated peptides. BMC Immunol..

[63] Keith B., Simon M.C. (2007). Hypoxia-inducible factors, stem cells, and cancer. Cell.

[64] Heddleston J.M., Li Z., Lathia J.D., Bao S., Hjelmeland A.B., Rich J.N. (2010). Hypoxia inducible factors in cancer stem cells. Br. J. Cancer.

[65] Koumenis C. (2006). Er stress, hypoxia tolerance and tumor progression. Curr. Mol. Med..

[66] Kaelin W.G., Ratcliffe P.J. (2008). Oxygen sensing by metazoans: The central role of the hif hydroxylase pathway. Mol. Cell.

[67] Jiang D.D., Niwa M., Koong A.C. (2015). Targeting the ire1 alpha-xbp1 branch of the unfolded protein response in human diseases. Semin. Cancer Biol..

[68] Muz B., de la Puente P., Azab F., Azab A.K. (2015). The role of hypoxia in cancer progression, angiogenesis, metastasis, and resistance to therapy. Hypoxia.

[69] Nishida N., Yano H., Nishida T., Kamura T., Kojiro M. (2006). Angiogenesis in cancer. Vasc. Health Risk Manag..

[70] Koumenis C., Wouters B.G. (2006). “Translating” tumor hypoxia: Unfolded protein response (upr)-dependent and upr-independent pathways. Mol. Cancer Res..

[71] Moenner M., Pluquet O., Bouchecareilh M., Chevet E. (2007). Integrated endoplasmic reticulum stress responses in cancer. Cancer Res..

[72] Mujcic H., Rzymski T., Rouschop K.M.A., Koritzinsky M., Milani M., Harris A.L., Wouters B.G. (2009). Hypoxic activation of the unfolded protein response (upr) induces expression of the metastasis-associated gene lamp3. Radiother. Oncol..

[73] Wang Y.G., Alam G.N., Ning Y., Visioli F., Dong Z.H., Nor J.E., Polverini P.J. (2012). The unfolded protein response induces the angiogenic switch in human tumor cells through the perk/atf4 pathway. Cancer Res..

[74] Rouschop K.M.A., van den Beucken T., Dubois L., Niessen H., Bussink J., Savelkouls K., Keulers T., Mujcic H., Landuyt W., Voncken J.W. (2010). The unfolded protein response protects human tumor cells during hypoxia through regulation of the autophagy genes map1lc3b and atg5. J. Clin. Investig..

[75] Cojocari D., Vellanki R.N., Sit B., Uehling D., Koritzinsky M., Wouters B.G. (2013). New small molecule inhibitors of upr activation demonstrate that perk, but not ire1 alpha signaling is essential for promoting adaptation and survival to hypoxia. Radiother. Oncol..

[76] May D., Itin A., Gal O., Kalinski H., Feinstein E., Keshet E. (2005). Ero1-l alpha plays a key role in a hif-1-mediated pathway to improve disulfide bond formation and vegf secretion under hypoxia: Implication for cancer. Oncogene.

[77] Farina A.R., Cappabianca L., Sebastiano M., Zelli V., Guadagni S., Mackay A.R. (2020). Hypoxia-induced alternative splicing: The 11th hallmark of cancer. J. Exp. Clin. Cancer Res..

[78] Young R., Ackerman D., Quinn Z., Mancuso A., Gruber M., Liu L.P., Giannoukos D., Bobovnikova-Marjon E., Diehl J.A., Keith B. (2013). Dysregulated mTORC1 renders cells critically dependent on desaturated lipids for survival under tumor-like stress. Genes Dev..

[79] Semenza G.L., Nejfelt M.K., Chi S.M., Antonarakis S.E. (1991). Hypoxia-inducible nuclear factors bind to an enhancer element located 3′ to the human erythropoietin gene. Proc. Natl. Acad. Sci. USA.

[80] Wang G.L., Jiang B.H., Rue E.A., Semenza G.L. (1995). Hypoxia-inducible factor-1 is a basic-helix-loop-helix-pas heterodimer regulated by cellular o-2 tension. Proc. Natl. Acad. Sci. USA.

[81] Maxwell P.H., Wiesener M.S., Chang G.W., Clifford S.C., Vaux E.C., Cockman M.E., Wykoff C.C., Pugh C.W., Maher E.R., Ratcliffe P.J. (1999). The tumour suppressor protein vhl targets hypoxia-inducible factors for oxygen-dependent proteolysis. Nature.

[82] Ivan M., Kondo K., Yang H.F., Kim W., Valiando J., Ohh M., Salic A., Asara J.M., Lane W.S., Kaelin W.G. (2001). Hif alpha targeted for vhl-mediated destruction by proline hydroxylation: Implications for o-2 sensing. Science.

[83] Jaakkola P., Mole D.R., Tian Y.M., Wilson M.I., Gielbert J., Gaskell S.J., von Kriegsheim A., Hebestreit H.F., Mukherji M., Schofield C.J. (2001). Targeting of hif-alpha to the von hippel-lindau ubiquitylation complex by o-2-regulated prolyl hydroxylation. Science.

[84] Mahon P.C., Hirota K., Semenza G.L. (2001). Fih-1: A novel protein that interacts with hif-1alpha and vhl to mediate repression of hif-1 transcriptional activity. Genes Dev..

[85] Hu C.-J., Wang L.-Y., Chodosh L.A., Keith B., Simon M.C. (2003). Differential roles of hypoxia-inducible factor 1alpha (hif-1alpha) and hif-2alpha in hypoxic gene regulation. Mol. Cell. Biol..

[86] Seton-Rogers S. (2011). Hypoxia: Hif switch. Nat. Rev. Cancer.

[87] Semenza G.L. (2000). Hif-1: Using two hands to flip the angiogenic switch. Cancer Metastasis Rev..

[88] Koh M.Y., Powis G. (2012). Passing the baton: The hif switch. Trends Biochem. Sci..

[89] Bartoszewski R., Moszynska A., Serocki M., Cabaj A., Polten A., Ochocka R., Dell’Italia L., Bartoszewska S., Kroliczewski J., Dabrowski M. (2019). Primary endothelial cell-specific regulation of hypoxia-inducible factor (hif)-1 and hif-2 and their target gene expression profiles during hypoxia. FASEB J..

[90] Papandreou I., Cairns R.A., Fontana L., Lim A.L., Denko N.C. (2006). Hif-1 mediates adaptation to hypoxia by actively downregulating mitochondrial oxygen consumption. Cell Metab..

[91] Huang D., Li T.T., Li X.H., Zhang L., Sun L.C., He X.P., Zhong X.Y., Jia D.Y., Song L.B., Semenza G.L. (2014). Hif-1-mediated suppression of acyl-coa dehydrogenases and fatty acid oxidation is critical for cancer progression. Cell Rep..

[92] Kim J.W., Tchernyshyov I., Semenza G.L., Dang C.V. (2006). Hif-1-mediated expression of pyruvate dehydrogenase kinase: A metabolic switch required for cellular adaptation to hypoxia. Cell Metab..

[93] Wu P.F., Peters J.M., Harris R.A. (2001). Adaptive increase in pyruvate dehydrogenase kinase 4 during starvation is mediated by peroxisome proliferator-activated receptor alpha. Biochem. Biophys. Res. Commun..

[94] Fukuda R., Zhang H.F., Kim J.W., Shimoda L., Dang C.V., Semenza G.L. (2007). Hif-1 regulates cytochrome oxidase subunits to optimize efficiency of respiration in hypoxic cells. Cell.

[95] Chandel N.S., Maltepe E., Goldwasser E., Mathieu C.E., Simon M.C., Schumacker P.T. (1998). Mitochondrial reactive oxygen species trigger hypoxia-induced transcription. Proc. Natl. Acad. Sci. USA.

[96] Chiche J., Brahimi-Horn M.C., Pouyssegur J. (2010). Tumour hypoxia induces a metabolic shift causing acidosis: A common feature in cancer. J. Cell. Mol. Med..

[97] Chiche J., Ilc K., Laferriere J., Trottier E., Dayan F., Mazure N.M., Brahimi-Horn M.C., Pouyssegur J. (2009). Hypoxia-inducible carbonic anhydrase ix and xii promote tumor cell growth by counteracting acidosis through the regulation of the intracellular ph. Cancer Res..

[98] Chee N.T., Lohse I., Brothers S.P. (2019). Mrna-to-protein translation in hypoxia. Mol. Cancer.

[99] Thomas J.D., Dias L.M., Johannes G.J. (2008). Translational repression during chronic hypoxia is dependent on glucose levels. RNA.

[100] Fahling M. (2009). Surviving hypoxia by modulation of mrna translation rate. J. Cell. Mol. Med..

[101] Bellot G., Garcia-Medina R., Gounon P., Chiche J., Roux D., Pouyssegur J., Mazure N.M. (2009). Hypoxia-induced autophagy is mediated through hypoxia-inducible factor induction of bnip3 and bnip3l via their bh3 domains. Mol. Cell. Biol..

[102] Yi T.F., Papadopoulos E., Hagner P.R., Wagner G. (2013). Hypoxia-inducible factor-1 alpha (hif-1 alpha) promotes cap-dependent translation of selective mrnas through up-regulating initiation factor eif4e1 in breast cancer cells under hypoxia conditions. J. Biol. Chem..

[103] van den Beucken T., Magagnin M.G., Jutten B., Seigneuric R., Lambin P., Koritzinsky M., Wouters B.G. (2011). Translational control is a major contributor to hypoxia induced gene expression. Radiother. Oncol..

[104] Papandreou I., Lim A.L., Laderoute K., Denko N.C. (2008). Hypoxia signals autophagy in tumor cells via ampk activity, independent of hif-1, bnip3, and bnip3l. Cell Death Differ..

[105] Lodish H.F. (1997). Biogenesis of secretory proteins and cell surface receptors in mammalian cells: Posttranslational modifications and protein folding within the rough endoplasmic reticulum. Animal Cell Technology.

[106] Dong L., Krewson E.A., Yang L.V. (2017). Acidosis activates endoplasmic reticulum stress pathways through gpr4 in human vascular endothelial cells. Int. J. Mol. Sci..

[107] Maeyashiki C., Melhem H., Hering L., Baebler K., Cosin-Roger J., Schefer F., Weder B., Hausmann M., Scharl M., Rogler G. (2020). Activation of ph-sensing receptor ogr1 (gpr68) induces er stress via the ire1 alpha/jnk pathway in an intestinal epithelial cell model. Sci. Rep..

[108] Teixeira J., Basit F., Swarts H.G., Forkink M., Oliveira P.J., Willems P.H.G.M., Koopman W.J.H. (2018). Extracellular acidification induces ros- and mptp-mediated death in hek293 cells. Redox Biol..

[109] Denzel M.S., Antebi A. (2015). Hexosamine pathway and (er) protein quality control. Curr. Opin. Cell Biol..

[110] Moore C.E., Omikorede O., Gomez E., Willars G.B., Herbert T.P. (2011). Perk activation at low glucose concentration is mediated by serca pump inhibition and confers preemptive cytoprotection to pancreatic beta-cells. Mol. Endocrinol..

[111] Shimizu Y., Hendershot L.M. (2009). Oxidative folding: Cellular strategies for dealing with the resultant equimolar production of reactive oxygen species. Antioxid. Redox Signal..

[112] Liou G.Y., Storz P. (2010). Reactive oxygen species in cancer. Free Radic. Res..

[113] Verschoor M.L., Wilson L.A., Singh G. (2010). Mechanisms associated with mitochondrial-generated reactive oxygen species in cancer. Can. J. Physiol. Pharm..

[114] Gorlach A., Bertram K., Hudecova S., Krizanova O. (2015). Calcium and ros: A mutual interplay. Redox Biol..

[115] Melber A., Haynes C.M. (2018). Uprmt regulation and output: A stress response mediated by mitochondrial-nuclear communication. Cell Res..

[116] Kueh H.Y., Niethammer P., Mitchison T.J. (2013). Maintenance of mitochondrial oxygen homeostasis by cosubstrate compensation. Biophys. J..

[117] Shpilka T., Haynes C.M. (2018). The mitochondrial upr: Mechanisms, physiological functions and implications in ageing. Nat. Rev. Mol. Cell Biol..

[118] Munch C. (2018). The different axes of the mammalian mitochondrial unfolded protein response. BMC Biol..

[119] Ratcliffe P.J. (2007). Hif-1 and hif-2: Working alone or together in hypoxia?. J. Clin. Investig..

[120] Loboda A., Jozkowicz A., Dulak J. (2012). Hif-1 versus hif-2—is one more important than the other?. Vasc. Pharmacol..

[121] Raval R.R., Lau K.W., Tran M.G., Sowter H.M., Mandriota S.J., Li J.L., Pugh C.W., Maxwell P.H., Harris A.L., Ratcliffe P.J. (2005). Contrasting properties of hypoxia-inducible factor 1 (hif-1) and hif-2 in von hippel-lindau-associated renal cell carcinoma. Mol. Cell. Biol..

[122] Zhang P., Yao Q., Lu L., Li Y., Chen P.J., Duan C.M. (2014). Hypoxia-inducible factor 3 is an oxygen-dependent transcription activator and regulates a distinct transcriptional response to hypoxia. Cell Rep..

[123] Janaszak-Jasiecka A., Bartoszewska S., Kochan K., Piotrowski A., Kalinowski L., Kamysz W., Ochocka R.J., Bartoszewski R., Collawn J.F. (2016). Mir-429 regulates the transition between hypoxia-inducible factor (hif)1a and hif3a expression in human endothelial cells. Sci. Rep..

[124] Ravenna L., Salvatori L., Russo M.A. (2016). Hif3alpha: The little we know. FEBS J..

[125] Moszynska A., Jaskiewicz M., Serocki M., Cabaj A., Crossman D.K., Bartoszewska S., Gebert M., Dabrowski M., Collawn J.F., Bartoszewski R. (2022). The hypoxia-induced changes in mirna-mrna in rna-induced silencing complexes and hif-2 induced mirnas in human endothelial cells. FASEB J..

[126] Buchler P., Reber H.A., Buchler M., Shrinkante S., Buchler M.W., Friess H., Semenza G.L., Hines O.J. (2003). Hypoxia-inducible factor 1 regulates vascular endothelial growth factor expression in human pancreatic cancer. Pancreas.

[127] Schipani E., Ryan H.E., Didrickson S., Kobayashi T., Knight M., Johnson R.S. (2001). Hypoxia in cartilage: Hif-1 alpha is essential for chondrocyte growth arrest and survival. Genes Dev..

[128] Pichiule P., Chavez J.C., LaManna J.C. (2004). Hypoxic regulation of angiopoietin-2 expression in endothelial cells. J. Biol. Chem..

[129] Schito L., Rey S., Tafani M., Zhang H.F., Wong C.C.L., Russo A., Russo M.A., Semenza G.L. (2012). Hypoxia-inducible factor 1-dependent expression of platelet-derived growth factor b promotes lymphatic metastasis of hypoxic breast cancer cells. Proc. Natl. Acad. Sci. USA.

[130] Adelman D.M., Gertsenstein M., Nagy A., Simon M.C., Maltepe E. (2000). Placental cell fates are regulated in vivo by hif-mediated hypoxia responses. Genes Dev..

[131] Han Z.-B., Ren H., Zhao H., Chi Y., Chen K., Zhou B., Liu Y.-j., Zhang L., Xu B., Liu B. (2008). Hypoxia-inducible factor (hif)-1α directly enhances the transcriptional activity of stem cell factor (scf) in response to hypoxia and epidermal growth factor (egf). Carcinogenesis.

[132] Benita Y., Kikuchi H., Smith A.D., Zhang M.Q., Chung D.C., Xavier R.J. (2009). An integrative genomics approach identifies hypoxia inducible factor-1 (hif-1)-target genes that form the core response to hypoxia. Nucleic Acids Res..

[133] Haase V.H. (2013). Regulation of erythropoiesis by hypoxia-inducible factors. Blood Rev..

[134] Kirito K. (2011). [Regulation of erythropoiesis by hypoxia inducible factors (hifs)]. [Rinsho Ketsueki] Jpn. J. Clin. Hematol..

[135] Semenza G.L., Wang G.L. (1992). A nuclear factor induced by hypoxia via de novo protein synthesis binds to the human erythropoietin gene enhancer at a site required for transcriptional activation. Mol. Cell. Biol..

[136] Shah Y.M., Matsubara T., Ito S., Yim S.H., Gonzalez F.J. (2009). Intestinal hypoxia-inducible transcription factors are essential for iron absorption following iron deficiency. Cell Metab..

[137] Peyssonnaux C., Zinkernagel A.S., Schuepbach R.A., Rankin E., Vaulont S., Haase V.H., Nizet V., Johnson R.S. (2007). Regulation of iron homeostasis by the hypoxia-inducible transcription factors (hifs). J. Clin. Investig..

[138] Schroder M., Kaufman R.J. (2005). The mammalian unfolded protein response. Annu. Rev. Biochem..

[139] Ye J., Rawson R.B., Komuro R., Chen X., Dave U.P., Prywes R., Brown M.S., Goldstein J.L. (2000). Er stress induces cleavage of membrane-bound atf6 by the same proteases that process srebps. Mol. Cell.

[140] Haze K., Yoshida H., Yanagi H., Yura T., Mori K. (1999). Mammalian transcription factor atf6 is synthesized as a transmembrane protein and activated by proteolysis in response to endoplasmic reticulum stress. Mol. Biol. Cell.

[141] Bartoszewski R., Gebert M., Janaszak-Jasiecka A., Cabaj A., Kroliczewski J., Bartoszewska S., Sobolewska A., Crossman D.K., Ochocka R., Kamysz W. (2020). Genome-wide mrna profiling identifies rcan1 and gadd45a as regulators of the transitional switch from survival to apoptosis during er stress. FEBS J..

[142] Li M.Q., Baumeister P., Roy B., Phan T., Foti D., Luo S.Z., Lee A.S. (2000). Atf6 as a transcription activator of the endoplasmic reticulum stress element: Thapsigargin stress-induced changes and synergistic interactions with nf-y and yy1. Mol. Cell. Biol..

[143] Zhang K.Z., Kaufman R.J. (2004). Signaling the unfolded protein response from the endoplasmic reticulum. J. Biol. Chem..

[144] Han D., Lerner A.G., Vande Walle L., Upton J.P., Xu W.H., Hagen A., Backes B.J., Oakes S.A., Papa F.R. (2009). Ire1 alpha kinase activation modes control alternate endoribonuclease outputs to determine divergent cell fates. Cell.

[145] Maurel M., Chevet E., Tavernier J., Gerlo S. (2014). Getting ridd of rna: Ire1 in cell fate regulation. Trends Biochem. Sci..

[146] Chen X., Iliopoulos D., Zhang Q., Tang Q.Z., Greenblatt M.B., Hatziapostolou M., Lim E., Tam W.L., Ni M., Chen Y.W. (2014). Xbp1 promotes triple-negative breast cancer by controlling the hif1 alpha pathway. Nature.

[147] Almanza A., Mnich K., Blomme A., Robinson C.M., Rodriguez-Blanco G., Kierszniowska S., McGrath E.P., Le Gallo M., Pilalis E., Swinnen J.V. (2022). Regulated ire1 alpha-dependent decay (ridd)-mediated reprograming of lipid metabolism in cancer. Nat. Commun..

[148] Yoshida H., Matsui T., Yamamoto A., Okada T., Mori K. (2001). Xbp1 mrna is induced by atf6 and spliced by ire1 in response to er stress to produce a highly active transcription factor. Cell.

[149] Gebert M., Sobolewska A., Bartoszewska S., Cabaj A., Crossman D.K., Madanecki P., Dabrowski M., Collawn J.F., Bartoszewski R., Kroliczewski J. (2021). Genome-wide mrna profiling identifies x-box-binding protein 1 (xbp1) as an ire1 and puma repressor. Cell. Mol. Life Sci..

[150] Gonen N., Sabath N., Burge C.B., Shalgi R. (2019). Widespread perk-dependent repression of er targets in response to er stress. Sci. Rep..

[151] Han J., Backa S.H., Hur J., Lin Y.H., Gildersleeve R., Shan J.X., Yuan C.L., Krokowski D., Wang S.Y., Hatzoglou M. (2013). Er-stress-induced transcriptional regulation increases protein synthesis leading to cell death. Nat. Cell Biol..

[152] Rutkowski D.T., Kaufman R.J. (2003). All roads lead to atf4. Dev. Cell.

[153] Novoa I., Zeng H.Q., Harding H.P., Ron D. (2001). Feedback inhibition of the unfolded protein response by gadd34-mediated dephosphorylation of eif2 alpha. J. Cell Biol..

[154] Urano F., Wang X.Z., Bertolotti A., Zhang Y.H., Chung P., Harding H.P., Ron D. (2000). Coupling of stress in the er to activation of jnk protein kinases by transmembrane protein kinase ire1. Science.

[155] Iurlaro R., Munoz-Pinedo C. (2016). Cell death induced by endoplasmic reticulum stress. FEBS J..

[156] Reimertz C., Kogel D., Rami A., Chittenden T., Prehn J.H.M. (2003). Gene expression during er stress-induced apoptosis in neurons: Induction of the bh3-only protein bbc3/puma and activation of the mitochondrial apoptosis pathway. J. Cell Biol..

[157] Gupta S., Giricz Z., Natoni A., Donnelly N., Deegan S., Szegezdi E., Samali A. (2012). Noxa contributes to the sensitivity of perk-deficient cells to er stress. FEBS Lett..

[158] Rosebeck S., Sudini K., Chen T.N., Leaman D.W. (2011). Involvement of noxa in mediating cellular er stress responses to lytic virus infection. Virology.

[159] Wang Q., Mora-Jensen H., Weniger M.A., Perez-Galan P., Wolford C., Hai T., Ron D., Chen W.P., Trenkle W., Wiestner A. (2009). Erad inhibitors integrate er stress with an epigenetic mechanism to activate bh3-only protein noxa in cancer cells. Proc. Natl. Acad. Sci. USA.

[160] Mukherji S., Ebert M.S., Zheng G.X., Tsang J.S., Sharp P.A., van Oudenaarden A. (2011). Micrornas can generate thresholds in target gene expression. Nat. Genet..

[161] Byrd A., Brewer J. (2011). Microrna-mediated repression of xbp1: A novel mechanism for regulation of a upr transcriptional activator. J. Immunol..

[162] Cheung O., Mirshahi F., Min H., Zhou H., Fuchs M., Sanyal A.J. (2008). Silencing microrna mir-34a and 451 promotes recovery from unfolded protein response (upr) and reverses nonalcoholic fatty liver disease (nafld). Hepatology.

[163] Bartoszewska S., Kochan K., Madanecki P., Piotrowski A., Ochocka R., Collawn J.F., Bartoszewski R. (2013). Regulation of the unfolded protein response by micrornas. Cell. Mol. Biol. Lett..

[164] Oyadomari S., Mori M. (2003). Roles of chop/gadd153 in endoplasmic reticulum stress. Cell Death Differ..

[165] B’chir W., Maurin A.C., Carraro V., Averous J., Jousse C., Muranishi Y., Parry L., Stepien G., Fafournoux P., Bruhat A. (2013). The eif2 alpha/atf4 pathway is essential for stress-induced autophagy gene expression. Nucleic Acids Res..

[166] Saveljeva S., Mc Laughlin S.L., Vandenabeele P., Samali A., Bertrand M.J. (2015). Endoplasmic reticulum stress induces ligand-independent tnfr1-mediated necroptosis in l929 cells. Cell Death Dis..

[167] Livezey M., Huang R., Hergenrother P.J., Shapiro D.J. (2018). Strong and sustained activation of the anticipatory unfolded protein response induces necrotic cell death. Cell Death Differ..

[168] Shirjang S., Mansoori B., Asghari S., Duijf P.H.G., Mohammadi A., Gjerstorff M., Baradaran B. (2019). Micrornas in cancer cell death pathways: Apoptosis and necroptosis. Free Radic. Biol. Med..

[169] Kishino A., Hayashi K., Maeda M., Jike T., Hidai C., Nomura Y., Oshima T. (2019). Caspase-8 regulates endoplasmic reticulum stress-induced necroptosis independent of the apoptosis pathway in auditory cells. Int. J. Mol. Sci..

[170] Ding B.X., Parmigiani A., Divakaruni A.S., Archer K., Murphy A.N., Budanov A.V. (2016). Sestrin2 is induced by glucose starvation via the unfolded protein response and protects cells from non-canonical necroptotic cell death. Sci. Rep..

[171] Cheng S.B., Nakashima A., Huber W.J., Davis S., Banerjee S., Huang Z.P., Saito S., Sadovsky Y., Sharma S. (2019). Pyroptosis is a critical inflammatory pathway in the placenta from early onset preeclampsia and in human trophoblasts exposed to hypoxia and endoplasmic reticulum stressors. Cell Death Dis..

[172] Yang Z., Wang Y., Zhang Y., He X., Zhong C.Q., Ni H., Chen X., Liang Y., Wu J., Zhao S. (2018). Rip3 targets pyruvate dehydrogenase complex to increase aerobic respiration in tnf-induced necroptosis. Nat. Cell Biol..

[173] Qiu X.F., Zhang Y.Y., Han J.H. (2018). Rip3 is an upregulator of aerobic metabolism and the enhanced respiration by necrosomal rip3 feeds back on necrosome to promote necroptosis. Cell Death Differ..

[174] Fulda S. (2013). Alternative cell death pathways and cell metabolism. Int. J. Cell Biol..

[175] Gong Y., Fan Z., Luo G., Yang C., Huang Q., Fan K., Cheng H., Jin K., Ni Q., Yu X. (2019). The role of necroptosis in cancer biology and therapy. Mol. Cancer.

[176] Babour A., Bicknell A.A., Tourtellotte J., Niwa M. (2010). A surveillance pathway monitors the fitness of the endoplasmic reticulum to control its inheritance. Cell.

[177] Sun M.Y., Ma D.S., Zhao S., Wang L., Ma C.Y., Bai Y. (2018). Salidroside mitigates hypoxia/reoxygenation injury by alleviating endoplasmic reticulum stress-induced apoptosis in h9c2 cardiomyocytes. Mol. Med. Rep..

[178] Tameire F., Verginadis I.I., Koumenis C. (2015). Cell intrinsic and extrinsic activators of the unfolded protein response in cancer: Mechanisms and targets for therapy. Semin. Cancer Biol..

[179] Delbrel E., Soumare A., Naguez A., Label R., Bernard O., Bruhat A., Fafournoux P., Tremblais G., Marchant D., Gille T. (2018). Hif-1 alpha triggers er stress and chop-mediated apoptosis in alveolar epithelial cells, a key event in pulmonary fibrosis. Sci. Rep..

[180] Blazanin N., Son J., Craig-Lucas A.B., John C.L., Breech K.J., Podolsky M.A., Glick A.B. (2017). Er stress and distinct outputs of the ire1 alpha rnase control proliferation and senescence in response to oncogenic ras. Proc. Natl. Acad. Sci. USA.

[181] Martin J. (1997). Molecular chaperones and mitochondrial protein folding. J. Bioenerg. Biomembr..

[182] Baker B.M., Nargund A.M., Sun T., Haynes C.M. (2012). Protective coupling of mitochondrial function and protein synthesis via the eif2 alpha kinase gcn-2. PLoS Genet..

[183] Sun L.L., Chen C.M., Zhang J., Wang J., Yang C.Z., Lin L.Z. (2019). Glucose-regulated protein 78 signaling regulates hypoxia-induced epithelial-mesenchymal transition in a549 cells. Front. Oncol..

[184] Scheuner D., Song B.B., McEwen E., Liu C., Laybutt R., Gillespie P., Saunders T., Bonner-Weir S., Kaufman R.J. (2001). Translational control is required for the unfolded protein response and in vivo glucose homeostasis. Mol. Cell.

[185] Koong A.C., Auger E.A., Chen E.Y., Giaccia A.J. (1994). The regulation of grp78 and messenger-rna levels by hypoxia is modulated by protein-kinase-c activators and inhibitors. Radiat. Res..

[186] Raiter A., Weiss C., Bechor Z., Ben-Dor I., Battler A., Kaplan B., Hardy B. (2010). Activation of grp78 on endothelial cell membranes by an adam15-derived peptide induces angiogenesis. J. Vasc. Res..

[187] Koong A.C., Chen E.Y., Lee A.S., Brown J.M., Giaccia A.J. (1994). Increased cytotoxicity of chronic hypoxic cells by molecular inhibition of grp78 induction. Int. J. Radiat. Oncol..

[188] Koumenis C., Naczki C., Koritzinsky M., Rastani S., Diehl A., Sonenberg N., Koromilas A., Wouters B.G. (2002). Regulation of protein synthesis by hypoxia via activation of the endoplasmic reticulum kinase perk and phosphorylation of the translation initiation factor eif2alpha. Mol. Cell. Biol..

[189] Blais J.D., Filipenko V., Bi M.X., Harding H.P., Ron D., Koumenis C., Wouters B.G., Bell J.C. (2004). Activating transcription factor 4 is translationally regulated by hypoxic stress. Mol. Cell. Biol..

[190] Liu L.P., Cash T.P., Jones R.G., Keith B., Thompson C.B., Simon M.C. (2006). Hypoxia-induced energy stress regulates mrna translation and cell growth. Mol. Cell.

[191] Ye J., Koumenis C. (2009). Atf4, an er stress and hypoxia-inducible transcription factor and its potential role in hypoxia tolerance and tumorigenesis. Curr. Mol. Med..

[192] Bensellam M., Maxwell E., Jonas J.C., Chan J., Laybutt D.R. (2015). Hypoxia induces beta cell death by inhibiting the adaptive upr. Diabetologia.

[193] Banach A., Jiang Y.P., Roth E., Kuscu C., Cao J., Lin R.Z. (2019). Cemip upregulates bip to promote breast cancer cell survival in hypoxia. Oncotarget.

[194] Fawcett T.W., Martindale J.L., Guyton K.Z., Hai T., Holbrook N.J. (1999). Complexes containing activating transcription factor (atf)/camp-responsive-element-binding protein (creb) interact with the ccaat enhancer-binding protein (c/ebp)-atf composite site to regulate gadd153 expression during the stress response. Biochem. J..

[195] Wolfgang C.D., Chen B.P.C., Martindale J.L., Holbrook N.J., Hai T. (1997). Gadd153/chop10, a potential target gene of the transcriptional repressor atf3. Mol. Cell. Biol..

[196] Rouschop K.M., Dubois L.J., Keulers T.G., van den Beucken T., Lambin P., Bussink J., van der Kogel A.J., Koritzinsky M., Wouters B.G. (2013). Perk/eif2alpha signaling protects therapy resistant hypoxic cells through induction of glutathione synthesis and protection against ros. Proc. Natl. Acad. Sci. USA.

[197] Mujcic H., Nagelkerke A., Rouschop K.M.A., Chung S., Chaudary N., Span P.N., Clarke B., Milosevic M., Sykes J., Hill R.P. (2013). Hypoxic activation of the perk/eif2 alpha arm of the unfolded protein response promotes metastasis through induction of lamp3. Clin. Cancer Res..

[198] Saxena K., Jolly M.K. (2019). Acute vs. Chronic vs. Cyclic hypoxia: Their differential dynamics, molecular mechanisms, and effects on tumor progression. Biomolecules.

[199] Chen A., Sceneay J., Godde N., Kinwel T., Ham S., Thompson E.W., Humbert P.O., Moller A. (2018). Intermittent hypoxia induces a metastatic phenotype in breast cancer. Oncogene.

[200] Kochan-Jamrozy K., Kroliczewski J., Moszynska A., Collawn J.F., Bartoszewski R. (2019). Mirna networks modulate human endothelial cell adaptation to cyclic hypoxia. Cell. Signal..

[201] Ivanova I.G., Park C.V., Yemm A.I., Kenneth N.S. (2018). Perk/eif2 alpha signaling inhibits hif-induced gene expression during the unfolded protein response via yb1-dependent regulation of hif1 alpha translation. Nucleic Acids Res..

[202] Hiwatashi Y., Kanno K., Takasaki C., Goryo K., Sato T., Torii S., Sogawa K., Yasumoto K. (2011). Phd1 interacts with atf4 and negatively regulates its transcriptional activity without prolyl hydroxylation. Exp. Cell Res..

[203] van den Beucken T., Koritzinsky M., Niessen H., Dubois L., Savelkouls K., Mujcic H., Jutten B., Kopacek J., Pastorekova S., van der Kogel A.J. (2009). Hypoxia-induced expression of carbonic anhydrase 9 is dependent on the unfolded protein response. J. Biol. Chem..

[204] Singleton D.C., Harris A.L. (2012). Targeting the atf4 pathway in cancer therapy. Expert Opin. Ther. Targets.

[205] Rzymski T., Milani M., Pike L., Buffa F., Mellor H.R., Winchester L., Pires I., Hammond E., Ragoussis I., Harris A.L. (2010). Regulation of autophagy by atf4 in response to severe hypoxia. Oncogene.

[206] Mudassar F., Shen H., O’Neill G., Hau E. (2020). Targeting tumor hypoxia and mitochondrial metabolism with anti-parasitic drugs to improve radiation response in high-grade gliomas. J. Exp. Clin. Cancer Res..

[207] Rozpedek W., Pytel D., Mucha B., Leszczynska H., Diehl J.A., Majsterek I. (2016). The role of the perk/eif2 alpha/atf4/chop signaling pathway in tumor progression during endoplasmic reticulum stress. Curr. Mol. Med..

[208] Yang D., Gao L.L., Wang T.F., Qiao Z.D., Liang Y.J., Zhang P. (2014). Hypoxia triggers endothelial endoplasmic reticulum stress and apoptosis via induction of vldl receptor. FEBS Lett..

[209] Xie P., Duan Y.C., Guo X.Z., Hu L.N., Yu M.H. (2015). Sala attenuates hypoxia-induced endothelial endoplasmic reticulum stress and apoptosis via down-regulation of vldl receptor expression. Cell. Physiol. Biochem..

[210] Loinard C., Zouggari Y., Rueda P., Ramkhelawon B., Cochain C., Vilar J., Recalde A., Richart A., Charue D., Duriez M. (2012). C/ebp homologous protein-10 (chop-10) limits postnatal neovascularization through control of endothelial nitric oxide synthase gene expression. Circulation.

[211] De Pascali F., Hemann C., Samons K., Chen C.A., Zweier J.L. (2014). Hypoxia and reoxygenation induce endothelial nitric oxide synthase uncoupling in endothelial cells through tetrahydrobiopterin depletion and s-glutathionylation. Biochemistry.

[212] Badran M., Abuyassin B., Golbidi S., Ayas N., Laher I. (2016). Uncoupling of vascular nitric oxide synthase caused by intermittent hypoxia. Oxid. Med. Cell Longev..

[213] Jeong K., Kim K., Kim H., Oh Y., Kim S.J., Jo Y., Choe W. (2015). Hypoxia induces cyclophilin b through the activation of transcription factor 6 in gastric adeno carcinoma cells. Oncol. Lett..

[214] Sicari D., Fantuz M., Bellazzo A., Valentino E., Apollonio M., Pontisso I., Di Cristino F., Dal Ferro M., Bicciato S., Del Salf G. (2019). Mutant p53 improves cancer cells’ resistance to endoplasmic reticulum stress by sustaining activation of the upr regulator atf6. Oncogene.

[215] Liang H., Zhou Z., Chen C. (2018). Abstract 2042: Hypoxia induces mir-153 through the ire1α-xbp1 pathway to fine-tune the hif1α/vegfa axis in breast cancer angiogenesis. Cancer Res..

[216] Liang H., Xiao J., Zhou Z., Wu J., Ge F., Li Z., Zhang H., Sun J., Li F., Liu R. (2018). Hypoxia induces mir-153 through the ire1alpha-xbp1 pathway to fine tune the hif1alpha/vegfa axis in breast cancer angiogenesis. Oncogene.

[217] Xu X.D., Qimuge A.D., Wang H.L., Xing C., Gu Y., Liu S.S., Xu H., Hu M.R., Song L. (2017). Ire1 alpha/xbp1s branch of upr links hif1 alpha activation to mediate angii-dependent endothelial dysfunction under particulate matter (pm) 2.5 exposure. Sci. Rep..

[218] Romero-Ramirez L., Cao H., Nelson D., Hammond E., Lee A.H., Yoshida H., Mori K., Glimcher L.H., Denko N.C., Giaccia A.J. (2004). Xbp1 is essential for survival under hypoxic conditions and is required for tumor growth. Cancer Res..

[219] Romero L., Cao H., Hammond E., Giaccia A.J., Le Q.T., Koong A.C. (2004). Xbp1 is essential for survival under hypoxic conditions and is required for tumor growth. Int. J. Radiat. Oncol..

[220] Bouchecareilh M., Chevet E., Bikfalvi A., Moenner M., Drogat B., Auguste P., Nguyen D.T., Pineau R., Nalbantoglu J., Kaufman R. (2007). Ire1 signaling is essential for ischemia-induced vascular endothelial growth factor-a expression and contributes to angiogenesis and tumor growth in vivo. B Cancer.

[221] Drogat B., Auguste P., Nguyen D.T., Bouchecareilh M., Pineau R., Nalbantoglu J., Kaufman R.J., Chevet E., Bikfalvi A., Moenner M. (2007). Ire1 signaling is essential for ischemia-induced vascular endothelial growth factor-a expression and contributes to angiogenesis and tumor growth in vivo. Cancer Res..

[222] Karar J., Dolt K.S., Pasha M.A.Q. (2008). Endoplasmic reticulum stress response in murine kidney exposed to acute hypobaric hypoxia. FEBS Lett..

[223] Moszyńska A., Collawn J.F., Bartoszewski R. (2020). Ire1 endoribonuclease activity modulates hypoxic hif-1α signaling in human endothelial cells. Biomolecules.

[224] Cao X., He Y., Li X., Xu Y., Liu X. (2019). The ire1α-xbp1 pathway function in hypoxia-induced pulmonary vascular remodeling, is upregulated by quercetin, inhibits apoptosis and partially reverses the effect of quercetin in pasmcs. Am. J. Transl. Res..

[225] Duan Q.L., Chen C., Yang L., Li N., Gong W., Li S., Wang D.W. (2015). Microrna regulation of unfolded protein response transcription factor xbp1 in the progression of cardiac hypertrophy and heart failure in vivo. J. Transl. Med..

[226] Brewer J.W., Jackson K.P., Lee E.H., Smith K.M. (2018). The unfolded protein response, microrna-214, and expression of the transcription factor xbp1. J. Immunol..

[227] Chitnis N., Pytel D., Diehl J.A. (2013). Upr-inducible mirnas contribute to stressful situations. Trends Biochem. Sci..

[228] Maurel M., Chevet E. (2013). Endoplasmic reticulum stress signaling: The microrna connection. Am. J. Physiol. Cell Physiol..

[229] Karali E., Bellou S., Stellas D., Klinakis A., Murphy C., Fotsis T. (2014). Vegf signals through atf6 and perk to promote endothelial cell survival and angiogenesis in the absence of er stress. Mol. Cell.

[230] Urra H., Hetz C. (2014). A novel er stress-independent function of the upr in angiogenesis. Mol. Cell.

[231] Ghosh R., Lipson K.L., Sargent K.E., Mercurio A.M., Hunt J.S., Ron D., Urano F. (2010). Transcriptional regulation of vegf-a by the unfolded protein response pathway. PLoS ONE.

[232] Pereira E.R., Liao N., Neale G.A., Hendershot L.M. (2010). Transcriptional and post-transcriptional regulation of proangiogenic factors by the unfolded protein response. PLoS ONE.

[233] Roybal C.N., Hunsaker L.A., Barbash O., Vander Jagt D.L., Abcouwer S.F. (2005). The oxidative stressor arsenite activates vascular endothelial growth factor mrna transcription by an atf4-dependent mechanism. J. Biol. Chem..

[234] Kyriakakis E., Philippova M., Joshi M.B., Pfaff D., Bochkov V., Afonyushkin T., Erne P., Resink T.J. (2010). T-cadherin attenuates the perk branch of the unfolded protein response and protects vascular endothelial cells from endoplasmic reticulum stress-induced apoptosis. Cell. Signal..

[235] Afonyushkin T., Oskolkova O.V., Philippova M., Resink T.J., Erne P., Binder B.R., Bochkov V.N. (2010). Oxidized phospholipids regulate expression of atf4 and vegf in endothelial cells via nrf2-dependent mechanism: Novel point of convergence between electrophilic and unfolded protein stress pathways. Arterioscler. Thromb. Vasc. Biol..

[236] Liu L., Qi X., Chen Z., Shaw L., Cai J., Smith L.H., Grant M.B., Boulton M.E. (2013). Targeting the ire1alpha/xbp1 and atf6 arms of the unfolded protein response enhances vegf blockade to prevent retinal and choroidal neovascularization. Am. J. Pathol..

[237] Longchamp A., Mirabella T., Arduini A., MacArthur M.R., Das A., Trevino-Villarreal J.H., Hine C., Ben-Sahra I., Knudsen N.H., Brace L.E. (2018). Amino acid restriction triggers angiogenesis via gcn2/atf4 regulation of vegf and h2s production. Cell.

[238] Terashima J., Tachikawa C., Kudo K., Habano W., Ozawa S. (2013). An aryl hydrocarbon receptor induces vegf expression through atf4 under glucose deprivation in hepg2. BMC Mol. Biol..

[239] Pollreisz A., Afonyushkin T., Oskolkova O.V., Gruber F., Bochkov V.N., Schmidt-Erfurth U. (2013). Retinal pigment epithelium cells produce vegf in response to oxidized phospholipids through mechanisms involving atf4 and protein kinase ck2. Exp. Eye Res..

[240] Chai L., Ling K., He X., Yang R. (2013). Expression of atf4 and vegf in chorionic villus tissue in early spontaneous abortion. Eur. J. Obstet. Gynecol. Reprod. Biol..

[241] Oskolkova O.V., Afonyushkin T., Leitner A., von Schlieffen E., Gargalovic P.S., Lusis A.J., Binder B.R., Bochkov V.N. (2008). Atf4-dependent transcription is a key mechanism in vegf up-regulation by oxidized phospholipids: Critical role of oxidized sn-2 residues in activation of unfolded protein response. Blood.

[242] Pereira E.R., Frudd K., Awad W., Hendershot L.M. (2014). Endoplasmic reticulum (er) stress and hypoxia response pathways interact to potentiate hypoxia-inducible factor 1 (hif-1) transcriptional activity on targets like vascular endothelial growth factor (vegf). J. Biol. Chem..

[243] Semenza G.L. (2011). Hypoxia-inducible factor 1: Regulator of mitochondrial metabolism and mediator of ischemic preconditioning. Biochim. Biophys. Acta BBA-Mol. Cell Res..

[244] Sanjuan-Pla A., Cervera A.M., Apostolova N., Garcia-Bou R., Victor V.M., Murphy M.P., McCreath K.J. (2005). A targeted antioxidant reveals the importance of mitochondrial reactive oxygen species in the hypoxic signaling of hif-1 alpha. FEBS Lett..

[245] Guzy R.D., Hoyos B., Robin E., Chen H., Liu L.P., Mansfield K.D., Simon M.C., Hammerling U., Schumacker P.T. (2005). Mitochondrial complex iii is required for hypoxia-induced ros production and cellular oxygen sensing. Cell Metab..

[246] Brunelle J.K., Bell E.L., Quesada N.M., Vercauteren K., Tiranti V., Zeviani M., Scarpulla R.C., Chandel N.S. (2005). Oxygen sensing requires mitochondrial ros but not oxidative phosphorylation. Cell Metab..

[247] Song S., Tan J., Miao Y., Sun Z., Zhang Q. (2018). Intermittent-hypoxia-induced autophagy activation through the er-stress-related perk/eif2alpha/atf4 pathway is a protective response to pancreatic beta-cell apoptosis. Cell. Physiol. Biochem..

[248] Yang Y.Y., Shang J., Liu H.G. (2013). Role of endoplasmic reticular stress in aortic endothelial apoptosis induced by intermittent/persistent hypoxia. Chin. Med. J..

[249] Zweier J.L. (1988). Measurement of superoxide-derived free-radicals in the reperfused heart—evidence for a free-radical mechanism of reperfusion injury. J. Biol. Chem..

[250] Ambrosio G., Zweier J.L., Duilio C., Kuppusamy P., Santoro G., Elia P.P., Tritto I., Cirillo P., Condorelli M., Chiariello M. (1993). Evidence that mitochondrial respiration is a source of potentially toxic oxygen-free radicals in intact rabbit hearts subjected to ischemia and reflow. J. Biol. Chem..

[251] Imarisio C., Alchera E., Revanna C.B., Valente G., Follenzi A., Trisolini E., Boldorini R., Carini R. (2017). Oxidative and er stress-dependent ask1 activation in steatotic hepatocytes and kupffer cells sensitizes mice fatty liver to ischemia/reperfusion injury. Free Radic. Biol. Med..

[252] Van Kooten C., Pacchiarotta T., van der Pol P., de Fijter J., Schlagwein N., van Gijlswijk D., Mayboroda O. (2016). Er stress and loss of grp78 expression provides a link between renal ischemia/reperfusion injury and the urinary metabolome. Am. J. Transplant..

[253] Rao J., Yue S., Fu Y., Zhu J., Wang X., Busuttil R.W., Kupiec-Weglinski J.W., Lu L., Zhai Y. (2014). Atf6 mediates a pro-inflammatory synergy between er stress and tlr activation in the pathogenesis of liver ischemia-reperfusion injury. Am. J. Transplant..

[254] Gao F., Shen X., Lu T., Liu J., Busuttil R.W., Kupiec-Weglinski J.W., Zhai Y. (2012). Il-23 in liver ischemia/reperfusion injury (iri): A synergy between er stress and tlr4 activation. Am. J. Transplant..

[255] Balachandran P., Dubray B.J., Upadhya G.A., Jia J., Anderson C., Chapman W.D. (2011). Er stress is an important mediator of ischemia reperfusion injury in hepatocytes isolated from steatotic livers. Am. J. Transplant..

[256] Kaser A., Tomczak M., Blumberg R.S. (2011). “Er stress(ed out)!”: Paneth cells and ischemia-reperfusion injury of the small intestine. Gastroenterology.

[257] Ren F., Liu J., Gao F., Shen X.D., Busuttil R.W., Kupiec-Weglinski J.W., Zhai Y. (2010). Endoplasmic reticulum (er) stress modulates tissue inflammatory responses and its implication in liver ischemia/reperfusion injury (iri). Liver Transplant..

[258] Vilatoba M., Eckstein C., Ringland S., Bilbao G., Thompson A., Eckhoff D.E., Contreras J.L. (2005). Sodium 4-phenylbutyrate (pba) protects against liver ischemia reperfusion injury (i/r-injury) by inhibition of endoplasmic reticulum (er)-stress mediated apoptosis. Am. J. Transplant..

[259] Ricca L., Lecorche E., Hamelin J., Balducci G., Azoulay D., Lemoine A. (2014). The unfolded protein response (upr) can participate to the liver ischemic postconditioning protection against ischemia/reperfusion (i/r) injury via the modulation of nf-kb/chop/il-1 beta signaling pathway. Transpl. Int..

[260] Zhang C.C., He S.Q., Li Y.M., Li F., Liu Z.B., Liu J., Gong J.B. (2017). Bisoprolol protects myocardium cells against ischemia/reperfusion injury by attenuating unfolded protein response in rats. Sci. Rep..

[261] Le Pape S., Dimitrova E., Hannaert P., Konovalov A., Volmer R., Ron D., Thuillier R., Hauet T. (2014). Polynomial algebra reveals diverging roles of the unfolded protein response in endothelial cells during ischemia-reperfusion injury. FEBS Lett..

[262] Kim H., Zhao J., Lee D., Bai X., Cypel M., Keshavjee S., Liu M. (2014). Protein kinase c delta-mediated unfolded protein response and necrotic cell death contributes to ischemia-reperfusion induced injury in lung transplantation. J Heart Lung Transpl..

[263] Wang Z.V., Deng Y.F., Gao N.G., Pedrozo Z., Li D.L., Tan W., Liang N., Lehrman M.A., Rothermel B.A., Lee A.H. (2013). The unfolded protein response directly activates the hexosamine biosynthetic pathway to protect the heart from ischemia/reperfusion injury. Circulation.

[264] Li Y.P., Wang S.L., Liu B., Tang L., Kuang R.R., Wang X.B., Zhao C., Song X.D., Cao X.M., Wu X. (2016). Sulforaphane prevents rat cardiomyocytes from hypoxia/reoxygenation injury in vitro via activating sirt1 and subsequently inhibiting er stress. Acta Pharmacol. Sin..

[265] Xu J.Q., Hu H.X., Chen B., Yue R.C., Zhou Z., Liu Y., Zhang S., Xu L., Wang H., Yu Z.P. (2015). Lycopene protects against hypoxia/reoxygenation injury by alleviating er stress induced apoptosis in neonatal mouse cardiomyocytes. PLoS ONE.

[266] Guan G.P., Yang L., Huang W.Y., Zhang J., Zhang P.H., Yu H., Liu S.Y., Gu X. (2019). Mechanism of interactions between endoplasmic reticulum stress and autophagy in hypoxia/reoxygenation-induced injury of h9c2 cardiomyocytes. Mol. Med. Rep..

[267] Xing J., Xu H., Liu C.B., Wei Z.L., Wang Z.H., Zhao L., Ren L. (2019). Melatonin ameliorates endoplasmic reticulum stress in n2a neuroblastoma cell hypoxia-reoxygenation injury by activating the ampk-pak2 pathway. Cell Stress Chaperones.

[268] Li T., Chen L.L., Yu Y.Y., Yang B.B., Li P.Y., Tan X.Q. (2019). Resveratrol alleviates hypoxia/reoxygenation injury-induced mitochondrial oxidative stress in cardiomyocytes. Mol. Med. Rep..

[269] Deng T.M., Wang Y.H., Wang C.C., Yan H. (2019). Fabp4 silencing ameliorates hypoxia reoxygenation injury through the attenuation of endoplasmic reticulum stress-mediated apoptosis by activating pi3k/akt pathway. Life Sci..

[270] Xu Y.X., Wang W.T., Jin K.K., Zhu Q.F., Lin H.Z., Xie M.Y., Wang D.X. (2017). Perillyl alcohol protects human renal tubular epithelial cells from hypoxia/reoxygenation injury via inhibition of ros, endoplasmic reticulum stress and activation of pi3k/akt/enos pathway. Biomed. Pharmacother..

[271] Lei X., Zhang S., Hu H.X., Yue R.C., Wang H., Chen H.Y., Tan C.Y., Li K. (2014). Lycopene protects cardiomyocytes from hypoxia/reoxygenation injury via attenuating endoplasmic reticulum stress. J. Am. Coll. Cardiol..

[272] Wu X.D., Zhang Z.Y., Sun S., Li Y.Z., Wang X.R., Zhu X.Q., Li W.H., Liu X.H. (2013). Hypoxic preconditioning protects microvascular endothelial cells against hypoxia/reoxygenation injury by attenuating endoplasmic reticulum stress. Apoptosis.

[273] Samarasinghe D.A., Tapner M., Farrell G.C. (2000). Role of oxidative stress in hypoxia-reoxygenation injury to cultured rat hepatic sinusoidal endothelial cells. Hepatology.

[274] Samarasinghe D.A., Farrell G.C. (1996). Role of redox stress in hypoxia-reoxygenation injury to hepatic sinusoidal endothelial cells. Hepatology.

[275] Sakaki K., Kaufman R.J. (2013). Interaction between quality control systems for er protein folding and rna biogenesis. Worm.

[276] Araki K., Nagata K. (2012). Protein folding and quality control in the er. Cold Spring Harb. Perspect. Biol..

[277] Hebert D.N., Molinari M. (2007). In and out of the er: Protein folding, quality control, degradation, and related human diseases. Physiol. Rev..

[278] Listowski M.A., Heger E., Boguslawska D.M., Machnicka B., Kuliczkowski K., Leluk J., Sikorski A.F. (2013). Micrornas: Fine tuning of erythropoiesis. Cell. Mol. Biol. Lett..

[279] Schewe D.M., Aguirre-Ghiso J.A. (2008). Atf6 alpha-rheb-mtor signaling promotes survival of dormant tumor cells in vivo. Proc. Natl. Acad. Sci. USA.

[280] Ranganathan A.C., Zhang L., Adam A.P., Aguirre-Ghiso J.A. (2006). Functional coupling of p38-induced up-regulation of bip and activation of rna-dependent protein kinase-like endoplasmic reticulum kinase to drug resistance of dormant carcinoma cells. Cancer Res..

[281] Cox T.R., Rumney R.M.H., Schoof E.M., Perryman L., Hoye A.M., Agrawal A., Bird D., Ab Latif N., Forrest H., Evans H.R. (2015). The hypoxic cancer secretome induces pre-metastatic bone lesions through lysyl oxidase. Nature.

[282] Feng Y.X., Sokol E.S., Del Vecchio C.A., Sanduja S., Claessen J.H.L., Proia T.A., Jin D.X., Reinhardt F., Ploegh H.L., Wang Q. (2014). Epithelial-to-mesenchymal transition activates perk-eif2 alpha and sensitizes cells to endoplasmic reticulum stress. Cancer Discov..

[283] de Almeida S.F., Fleming J.V., Azevedo J.E., Carmo-Fonseca M., de Sousa M. (2007). Stimulation of an unfolded protein response impairs mhc class i expression. J. Immunol..

[284] Obiedat A., Seidel E., Mahameed M., Berhani O., Tsukerman P., Voutetakis K., Chatziioannou A., McMahon M., Avril T., Chevet E. (2019). Transcription of the nkg2d ligand mica is suppressed by the ire1/xbp1 pathway of the unfolded protein response through the regulation of e2f1. FASEB J..

[285] Logue S.E., McGrath E.P., Cleary P., Greene S., Mnich K., Almanza A., Chevet E., Dwyer R.M., Oommen A., Legembre P. (2018). Inhibition of ire1 rnase activity modulates the tumor cell secretome and enhances response to chemotherapy. Nat. Commun..

[286] Chopra S., Giovanelli P., Alvarado-Vazquez P.A., Alonso S., Song M., Sandoval T.A., Chae C.S., Tan C., Fonseca M.M., Gutierrez S. (2019). Ire1 alpha-xbp1 signaling in leukocytes controls prostaglandin biosynthesis and pain. Science.

[287] Thevenot P.T., Sierra R.A., Raber P.L., Al-Khami A.A., Trillo-Tinoco J., Zarreii P., Ochoa A.C., Cui Y., Del Valle L., Rodriguez P.C. (2014). The stress-response sensor chop regulates the function and accumulation of myeloid-derived suppressor cells in tumors. Immunity.

[288] Mohamed E., Sierra R.A., Trillo-Tinoco J., Cao Y., Innamarato P., Payne K.K., Pulido A.D., Mandula J., Zhang S.Z., Thevenot P. (2020). The unfolded protein response mediator perk governs myeloid cell-driven immunosuppression in tumors through inhibition of sting signaling. Immunity.

[289] Harnoss J.M., Le Thomas A., Reichelt M., Guttman O., Wu T.D., Marsters S.A., Shemorry A., Lawrence D.A., Kan D., Segal E. (2020). Ire1 alpha disruption in triple-negative breast cancer cooperates with antiangiogenic therapy by reversing er stress adaptation and remodeling the tumor microenvironment. Cancer Res..

[290] Bartoszewska S., Kochan K., Piotrowski A., Kamysz W., Ochocka R.J., Collawn J.F., Bartoszewski R. (2015). The hypoxia-inducible mir-429 regulates hypoxia-inducible factor-1alpha expression in human endothelial cells through a negative feedback loop. FASEB J..

[291] Scharping N.E., Menk A.V., Moreci R.S., Whetstone R.D., Dadey R.E., Watkins S.C., Ferris R.L., Delgoffe G.M. (2016). The tumor microenvironment represses t cell mitochondrial biogenesis to drive intratumoral t cell metabolic insufficiency and dysfunction. Immunity.

[292] Song M., Sandoval T.A., Chae C.S., Chopra S., Tan C., Rutkowski M.R., Raundhal M., Chaurio R.A., Payne K.K., Konrad C. (2018). Ire1 alpha-xbp1 controls t cell function in ovarian cancer by regulating mitochondrial activity. Nature.

[293] Hurst K.E., Lawrence K.A., Essman M.T., Walton Z.J., Leddy L.R., Thaxton J.E. (2019). Endoplasmic reticulum stress contributes to mitochondrial exhaustion of cd8(+) t cells. Cancer Immunol. Res..

[294] Bottcher J.P., Sousa C.R.E. (2018). The role of type 1 conventional dendritic cells in cancer immunity. Trends Cancer.

[295] Leung-Hagesteijn C., Erdmann N., Cheung G., Keats J.J., Stewart A.K., Reece D.E., Chung K.C., Tiedemann R.E. (2015). Xbp1s-negative tumor b cells and pre-plasmablasts mediate therapeutic proteasome inhibitor resistance in multiple myeloma (vol 24, pg 289, 2013). Cancer Cell.

[296] Evans S.M., Koch C.J. (2003). Prognostic significance of tumor oxygenation in humans. Cancer Lett..

[297] Le Q.T., Denko N.C., Giaccia A.J. (2004). Hypoxic gene expression and metastasis. Cancer Metast. Rev..

[298] Durand R.E., Aquino-Parsons C. (2001). Clinical relevance of intermittent tumour blood flow. Acta Oncol..

[299] Lanzen J., Braun R.D., Klitzman B., Brizel D., Secomb T.W., Dewhirst M.W. (2006). Direct demonstration of instabilities in oxygen concentrations within the extravascular compartment of an experimental tumor. Cancer Res..

[300] Koritzinsky M., Magagnin M.G., van den Beucken T., Seigneuric R., Savelkouls K., Dostie J., Pyronnet S., Kaufman R.J., Weppler S.A., Voncken J.W. (2006). Gene expression during acute and prolonged hypoxia is regulated by distinct mechanisms of translational control. EMBO J..

[301] Chaplin D.J., Hill S.A. (1995). Temporal heterogeneity in microregional erythrocyte flux in experimental solid tumors. Br. J. Cancer.

[302] Sutherland R.M., Ausserer W.A., Murphy B.J., Laderoute K.R. (1996). Tumor hypoxia and heterogeneity: Challenges and opportunities for the future. Semin. Radiat. Oncol..

[303] Mennerich D., Kubaichuk K., Raza G.S., Fuhrmann D.C., Herzig K.-H., Brüne B., Kietzmann T. (2022). Er-stress promotes vhl-independent degradation of hypoxia-inducible factors via fbxw1a/βtrcp. Redox Biol..

[304] Cardenas-Navia L.I., Yu D.H., Braun R.D., Brizel D.M., Secomb T.W., Dewhirst M.W. (2004). Tumor-dependent kinetics of partial pressure of oxygen fluctuations during air and oxygen breathing. Cancer Res..

[305] Janssen H.L.K., Haustermans K.M.G., Sprong D., Blommestijn G., Hofland I., Hoebers F.J., Blijweert E., Raleigh J.A., Semenza G.L., Varia M.A. (2002). Hif-1a, pimonidazole, and iododeoxyuridine to estimate hypoxia and perfusion in human head-and-neck tumors. Int. J. Radiat. Oncol..

[306] Bader S.B., Dewhirst M.W., Hammond E.M. (2021). Cyclic hypoxia: An update on its characteristics, methods to measure it and biological implications in cancer. Cancers.

[307] Zepeda A.B., Pessoa A., Castillo R.L., Figueroa C.A., Pulgar V.M., Farias J.G. (2013). Cellular and molecular mechanisms in the hypoxic tissue: Role of hif-1 and ros. Cell Biochem. Funct..

[308] Hsieh C.H., Shyu W.C., Chiang C.Y., Kuo J.W., Shen W.C., Liu R.S. (2011). Nadph oxidase subunit 4-mediated reactive oxygen species contribute to cycling hypoxia-promoted tumor progression in glioblastoma multiforme. PLoS ONE.

[309] Dewhirst M.W., Cao Y.T., Moeller B. (2008). Cycling hypoxia and free radicals regulate angiogenesis and radiotherapy response. Nat. Rev. Cancer.

[310] Almendros I., Martinez-Garcia M.A., Campos-Rodriguez F., Riveiro-Falkenbach E., Rodriguez-Peralto J.L., Nagore E., Martorell-Calatayud A., Blasco L.H., Roca J.B., Vives E.C. (2018). Intermittent hypoxia is associated with high hypoxia inducible factor-1 alpha but not high vascular endothelial growth factor cell expression in tumors of cutaneous melanoma patients. Front. Neurol..

[311] Yoon D.W., So D., Min S., Kim J., Lee M., Khalmuratova R., Cho C.H., Park J.W., Shin H.W. (2017). Accelerated tumor growth under intermittent hypoxia is associated with hypoxia-inducible factor-1-dependent adaptive responses to hypoxia. Oncotarget.

[312] Yoon D.W., Min S., Kim Y., Kim J.H., Lee G.Y., Lee M., Roza K., Park J.W., Shin H.W. (2017). Intermittent hypoxia promotes tumor growth in azoxymethane and dextran sodium sulfate-induced colon carcinogenesis mouse model. Sleep Med..

[313] Martinive P., Defresne F., Bouzin C., Saliez J., Lair F., Gregoire V., Michiels C., Dessy C., Feron O. (2008). Preconditioning of the tumor vasculature and tumor cells by intermittent hypoxia: Implications for anti-cancer therapies. Acta Clin. Belg..

[314] Franko A.J. (1981). Evidence against acute-hypoxia caused by intermittent blood-flow in emt6 and lewis lung-tumors. Radiat. Res..

[315] McKeown S.R. (2014). Defining normoxia, physoxia and hypoxia in tumours-implications for treatment response. Br. J. Radiol..

[316] Brazovskaja A., Treutlein B., Camp J.G. (2019). High-throughput single-cell transcriptomics on organoids. Curr. Opin. Biotechnol..

[317] Tekin H., Simmons S., Cummings B., Gao L.Y., Adiconis X., Hession C.C., Ghoshal A., Dionne D., Choudhury S.R., Yesilyurt V. (2018). Effects of 3d culturing conditions on the transcriptomic profile of stem-cell-derived neurons. Nat. Biomed. Eng..

[318] Kashima Y., Sakamoto Y., Kaneko K., Seki M., Suzuki Y., Suzuki A. (2020). Single-cell sequencing techniques from individual to multiomics analyses. Exp. Mol. Med..

[319] Stuart T., Satija R. (2019). Integrative single-cell analysis. Nat. Rev. Genet..

[320] Chen R.L., Forsyth N. (2019). Editorial: The development of new classes of hypoxia mimetic agents for clinical use. Front. Cell Dev. Biol..

[321] Guo M., Song L.P., Jiang Y., Liu W., Chen G.Q. (2006). Hypoxia-mimetic agents desferrioxamine and cobalt chloride induce leukemic cell apoptosis through different hypoxia-inducible factor-1 alpha independent mechanisms. Apoptosis.

[322] Bartoszewski R., Rab A., Fu L.W., Bartoszewska S., Collawn J., Bebok Z. (2011). Cftr expression regulation by the unfolded protein response. Method Enzymol..

[323] Lee H., Dey D.K., Kim K., Kim S., Kim E., Kang S.C., Bajpai V.K., Huh Y.S. (2022). Hypoxia-responsive nanomedicine to overcome tumor microenvironment-mediated resistance to chemo-photodynamic therapy. Mater. Today Adv..

[324] Roque N., Matias D., Balça-Silva J., Ferrer V.-P., Pessoa L.-S., Spohr T.-C.-L.-d.-S.-e. (2022). The interface of cancer, their microenvironment and nanotechnology. Oncologie.

[325] Cross B.C.S., Bond P.J., Sadowski P.G., Jha B.K., Zak J., Goodman J.M., Silverman R.H., Neubert T.A., Baxendale I.R., Ron D. (2012). The molecular basis for selective inhibition of unconventional mrna splicing by an ire1-binding small molecule. Proc. Natl. Acad. Sci. USA.

[326] Bartoszewska S., Kroliczewski J., Crossman D.K., Pogorzelska A., Baginski M., Collawn J.F., Bartoszewski R. (2021). Triazoloacridone c-1305 impairs xbp1 splicing by acting as a potential ire1alpha endoribonuclease inhibitor. Cell. Mol. Biol. Lett..

[327] Rabouw H.H., Langereis M.A., Anand A.A., Visser L.J., de Groot R.J., Walter P., van Kuppeveld F.J.M. (2019). Small molecule isrib suppresses the integrated stress response within a defined window of activation. Proc. Natl. Acad. Sci. USA.

[328] Gallagher C.M., Walter P. (2016). Ceapins inhibit atf6α signaling by selectively preventing transport of atf6α to the golgi apparatus during er stress. eLife.

[329] Bartoszewska S., Kamysz W., Jakiela B., Sanak M., Kroliczewski J., Bebok Z., Bartoszewski R., Collawn J.F. (2017). Mir-200b downregulates cftr during hypoxia in human lung epithelial cells. Cell. Mol. Biol. Lett..

[330] Sun S.M., Xuan F.J., Ge X.P., Zhu J., Zhang W.X. (2017). Dynamic mrna and mirna expression analysis in response to hypoxia and reoxygenation in the blunt snout bream (megalobrama amblycephala). Sci. Rep..

[331] Rupaimoole R., Wu S.Y., Pradeep S., Ivan C., Pecot C.V., Gharpure K.M., Nagaraja A.S., Armaiz-Pena G.N., McGuire M., Zand B. (2014). Hypoxia-mediated downregulation of mirna biogenesis promotes tumour progression. Nat. Commun..

[332] Rupaimoole R., Ivan C., Pecot C., Wu S., Pradeep S., Zand B., Nagaraja A., Gharpure K., Dalton H., Sadaoui N. (2014). Hypoxia is a master regulator of drosha- and dicer-dependent mirna biogenesis in cancer. Cancer Res..

[333] Zhao R.B., Qian L.J., Jiang L. (2014). Mirna-dependent cross-talk between vegf and ang-2 in hypoxia-induced microvascular dysfunction. Biochem. Biophys. Res. Commun..

[334] Hua Z., Lv Q., Ye W.B., Wong C.K.A., Cai G.P., Gu D.Y., Ji Y.H., Zhao C., Wang J.F., Yang B.B. (2006). Mirna-directed regulation of vegf and other angiogenic factors under hypoxia. PLoS ONE.

[335] Bartoszewski R., Sikorski A.F. (2018). Editorial focus: Entering into the non-coding rna era. Cell. Mol. Biol. Lett..

